# Superposition of Individual Activities: Urea-Mediated Suppression of Nitrate Uptake in the Dinoflagellate *Prorocentrum minimum* Revealed at the Population and Single-Cell Levels

**DOI:** 10.3389/fmicb.2016.01310

**Published:** 2016-08-25

**Authors:** Olga Matantseva, Sergei Skarlato, Angela Vogts, Ilya Pozdnyakov, Iris Liskow, Hendrik Schubert, Maren Voss

**Affiliations:** ^1^Institute of Cytology, Russian Academy of SciencesSt. Petersburg, Russia; ^2^Leibniz Institute for Baltic Sea ResearchWarnemünde, Rostock, Germany; ^3^Institute of Biological Sciences, University of RostockRostock, Germany

**Keywords:** dinoflagellates, heterogeneity, NanoSIMS, nitrate uptake, nitrogen assimilation, *Prorocentrum minimum*, single-cell research, urea uptake

## Abstract

Dinoflagellates readily use diverse inorganic and organic compounds as nitrogen sources, which is advantageous in eutrophied coastal areas exposed to high loads of anthropogenic nutrients, e.g., urea, one of the most abundant organic nitrogen substrates in seawater. Cell-to-cell variability in nutritional physiology can further enhance the diversity of metabolic strategies among dinoflagellates of the same species, but it has not been studied in free-living microalgae. We applied stable isotope tracers, isotope ratio mass spectrometry and nanoscale secondary ion mass spectrometry (NanoSIMS) to investigate the response of cultured nitrate-acclimated dinoflagellates *Prorocentrum minimum* to a sudden input of urea and the effect of urea on the concurrent nitrate uptake at the population and single-cell levels. We demonstrate that inputs of urea lead to suppression of nitrate uptake by *P. minimum*, and urea uptake exceeds the concurrent uptake of nitrate. Individual dinoflagellate cells within a population display significant heterogeneity in the rates of nutrient uptake and extent of the urea-mediated inhibition of the nitrate uptake, thus forming several groups characterized by different modes of nutrition. We conclude that urea originating from sporadic sources is rapidly utilized by dinoflagellates and can be used in biosynthesis or stored intracellularly depending on the nutrient status; therefore, sudden urea inputs can represent one of the factors triggering or supporting harmful algal blooms. Significant physiological heterogeneity revealed at the single-cell level is likely to play a role in alleviation of intra-population competition for resources and can affect the dynamics of phytoplankton populations and their maintenance in natural environments.

## Introduction

Unicellular photosynthetic eukaryotes play a crucial role in oceanic primary production, sequestration of inorganic carbon and biogeochemical cycling of macro- and micronutrients (Falkowski et al., 1998; Morel and Price, 2003; Chassot et al., 2010; Matantseva and Skarlato, 2013). For many years, interactions between microalgae and environment have been studied primarily at the population level. Although the information obtained by this traditional approach is invaluable, it cannot fully clarify the complexity of processes in microbial populations and communities. Fortunately, in the last decades many new methods for analysis at the single-cell level, such as microfluidics, single-cell sequencing, patch-clamping of microalgae, and nanoscale secondary ion mass spectrometry (NanoSIMS), were developed and applied in the field of environmental microbiology and ecophysiology (Brehm-Stecher and Johnson, 2004; Popa et al., 2007; Li et al., 2008; Musat et al., 2008, 2012; Pozdnyakov et al., 2014; Labonté et al., 2015; Martins and Locke, 2015). Currently NanoSIMS is probably the most powerful technique to study nutritional activity of individual cells. Nevertheless, so far only a limited number of works report usage of NanoSIMS in phytoplankton ecology and biogeochemistry research. Mostly, such works explore the symbiotic relationships, where microalgae act as symbionts or host organisms (Foster et al., 2011; Pernice et al., 2012, 2015; Kopp et al., 2013; Zehr, 2015), whereas nutritional heterogeneity within populations of microalgae remains largely ignored. However, numerous studies on bacteria and mammalian cellular lineages showed that variability among cells of the same population is ubiquitous and in many cases highly significant for the interpretation of bulk observations (Altschuler and Wu, 2010), which can also be true for populations of environmentally important protists.

Urea is one of the most common dissolved organic compounds in the marine environment. Partially it originates from natural sources, but significant proportion of urea enters coastal ecosystems due to anthropogenic pollution. Over the last decades, commercial urea production has dramatically increased to meet demands of agriculture and industry; therefore, the anthropogenic sources of urea have become extremely important (Glibert et al., 2006). Results of the field and laboratory research demonstrate that many phytoplankton organisms are able to utilize urea as a nitrogen source (Berman and Chava, 1999; Pustizzi et al., 2004; Solomon and Glibert, 2008; Sinclair et al., 2009). Among these organisms dinoflagellates attract special attention, because many of them cause harmful algal blooms (HABs), one of the major environmental problems of our days (Hallegraeff, 1993; Smayda, 1997; Sellner et al., 2003; Burkholder et al., 2006; Li et al., 2015). The urea uptake rates and urease activity in dinoflagellates can be very high and sufficient to cover their N requirements (Dyhrman and Anderson, 2003; Fan et al., 2003a,b; Solomon and Glibert, 2008; Jauzein et al., 2011; Hu et al., 2014; Ou et al., 2014). The ability of dinoflagellates to utilize urea is often linked to their success in eutrophied habitats. Moreover, it was assumed that elevated concentrations of urea in water may trigger initiation of dinoflagellate blooms (Glibert et al., 2001; Anderson et al., 2002; Heisler et al., 2008), although this correlation is not always observed.

Environmental concentrations of urea are generally low, as compared to nitrate which is the most abundant pool (ca. 88%) of bioavailable nitrogen in the ocean (Gruber, 2008). Nevertheless, urea can represent a preferential N source for dinoflagellates compared to nitrate (Fan et al., 2003a,b). Moreover, in coastal regions urea concentrations may occasionally exceed the concentrations of nitrate achieving levels as high as 50 μmol N l^−1^ (Lomas et al., 2002; Glibert et al., 2005; Switzer, 2008). Short-term elevations of urea content in water are usually caused by runoff from heavily fertilized regions and areas with open/damaged sewers, particularly after rainfalls and storms. Due to their transience, such events can be easily overlooked, despite monitoring efforts; at the same time, they may significantly affect the nutrient status of coastal and estuarine systems and precede large dinoflagellate blooms (Switzer, 2008).

Interpretation of the field and laboratory data on the urea uptake and assimilation by dinoflagellates is hampered by the scarcity of genomic information available for this group of organisms. Although urease activity in dinoflagellates has been studied for about 20 years (Solomon et al., 2010), the putative gene encoding urease in these eukaryotes was unknown until recently, when its sequence was identified in *Alexandrium tamarense* using transcriptomic information (Dagenais-Bellefeuille and Morse, 2013). At the same time, there is still no or little information concerning genes and proteins involved in nutrient transport in dinoflagellates. Transcriptomes of these microalgae are much smaller in size than their genomes and consequently easier to sequence. Sequencing of transcriptomes of several dinoflagellate species has been initiated within Marine Microbial Eukaryote Transcriptome Sequencing Project (MMETSP; http://data.imicrobe.us/project/view/104, Combined Assemblies; Keeling et al., 2014) in order to uncover molecular basics of dinoflagellate metabolism and cell biology. However, currently MMETSP provides sequenced transcriptomes, where sequences are not annotated. Therefore, special effort has to be undertaken to identify specific homologs by bioinformatical tools. The analysis of publicly available transcriptomic data is a promising starting point to gain fundamental knowledge on genes and proteins behind the N transport and assimilation in dinoflagellates and facilitate the interpretation of experimental data.

In this work, we studied how photosynthetic nitrate-acclimated dinoflagellates *Prorocentrum minimum* responded to a sudden urea input at the population level and how this net response was realized at the level of single cells. Simultaneously, we aimed to examine interactions between nitrate and urea uptake in these bloom-forming organisms and to obtain information on some key genes involved in uptake and assimilation of these substrates by screening publicly available *P. minimum* transcriptomes. Currently the information on heterogeneity within populations of dinoflagellates and molecular mechanisms of their metabolism is very scarce. Our work is aimed to fill this gap and promote detailed extensive research of nutrient consumption by microalgae at the single-cell and molecular levels.

## Materials and methods

### Culture material and growth conditions

We used the monoculture of dinoflagellates *P. minimum* (Pavillard) Schiller 1933, currently named *Prorocentrum cordatum* (Ostenfeld) Dodge 1975, from The Culture Collection of Algae and Protozoa, UK (CCAP clone 1136/16). The culture was grown at the Institute of Cytology RAS at salinity of 25o in artificial seawater-based f/2 medium (Guillard and Ryther, 1962) containing no silicate. All stock solutions as well as artificial sea water were sterilized by autoclaving or sterile filtration. The cultures were grown at 22–23°C and 100 μmol photons m^−2^ s^−1^ under a 12 h light: 12 h dark cycle. In order to minimize bacterial content and activity the mixture of bactericidal and bacteriostatic antibiotics (ampicillin and streptomycin) was added to the culture medium at the stage of pre-incubation (see Section Experimental Procedures).

### Experimental procedures

We used stable isotope tracers to study the concurrent uptake of urea and nitrate by nitrate-grown dinoflagellates and the effect of urea on the nitrate uptake. The experiments consisted of the two stages: (1) pre-incubation stage lasting for 7–10 d, and (2) incubation stage lasting for 2 h.

At the pre-incubation stage, the medium was inoculated with the *P. minimum* culture and nutrients (400 μmol l^−1^ sodium nitrate and 100 μmol l^−1^ monopotassium phosphate), and the culture was allowed to reach the cell density not less than 40 × 10^3^ cells ml^−1^ and the exponential growth phase. On mornings on which the incubation stage of the experiments was conducted, we roughly estimated concentration of nitrate in the experimental culture in order to add equal amount of urea-N as treatment.

**Figure 1:**
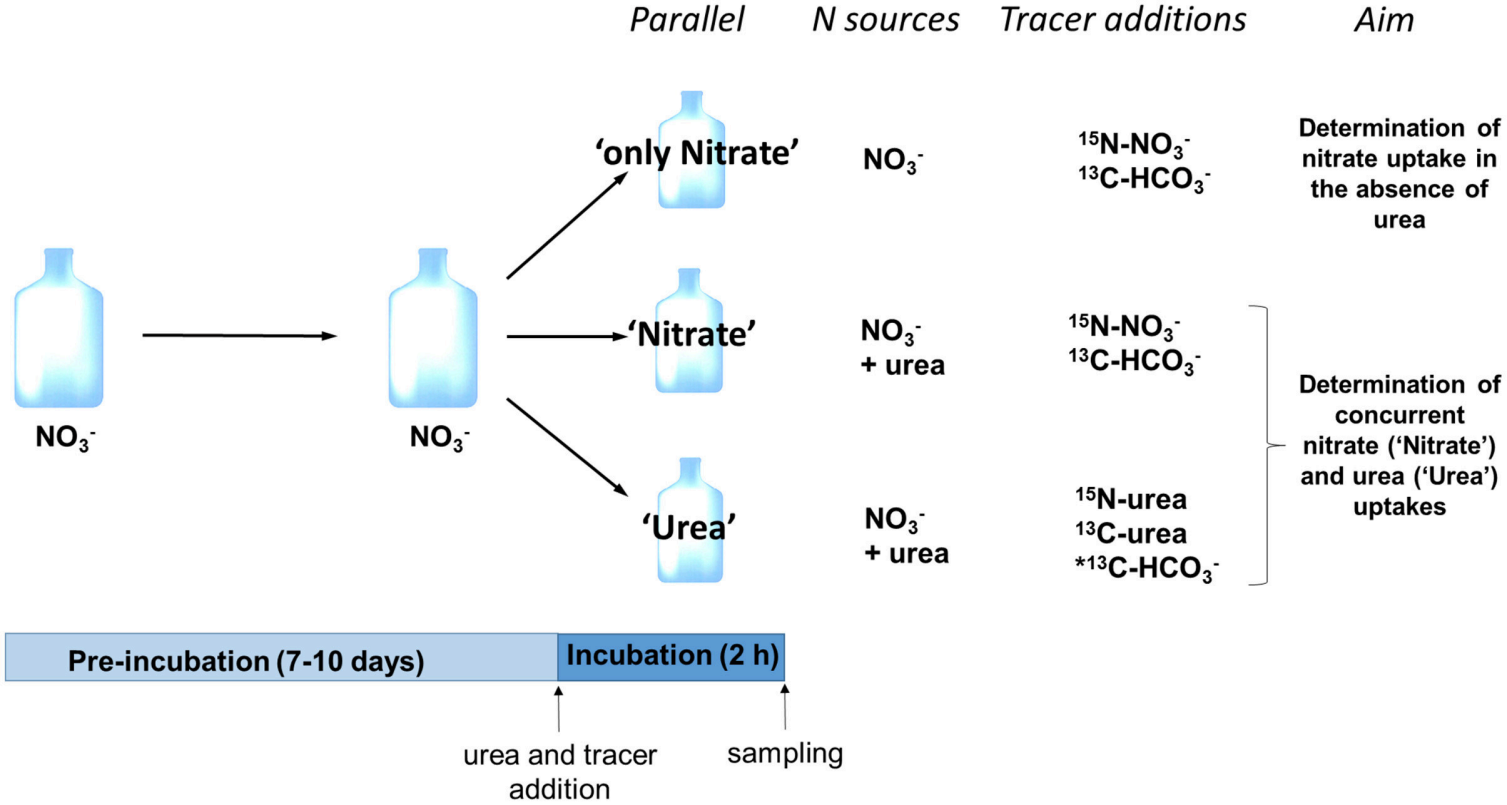
**Schematic representation of the experimental design**. A single experimental replicate is depicted; in total six experimental replicates were performed. Following pre-incubation with nitrate as a sole N source, three subcultures (parallels) were established; two of them received urea as a second N source (parallels “Nitrate” and “Urea”). Isotope tracers were added to measure nitrate uptake rate in the absence of urea (parallel “only Nitrate”), concurrent uptake rates of nitrate (parallel “Nitrate”) and urea (parallel “Urea”) and uptake rate of bicarbonate (all parallels). ^*^Note that in three replicates out of six, we added ^13^C-labled urea instead of ^13^C-bicarbonate to the parallel “Urea” in order to measure ^13^C-urea uptake.

At the incubation stage, the surface fraction of a culture growing on nitrate was split in three subcultures (further referred to as “parallels”) that allowed to measure the nitrate uptake in the absence of urea (parallel “only Nitrate”), nitrate uptake following the input of urea (parallel “Nitrate”), and uptake of newly added urea-N in the presence of nitrate (parallel “Urea”) (Figure 1). In addition, the carbon uptake (bicarbonate or urea-C) was determined in all parallels. Incubation was initiated by addition of urea (where appropriate) and stable isotope tracers (Figure 1) and lasted for 2 h in the middle of the light period. Urea was added to the subcultures “Urea” and “Nitrate” at urea-N concentration similar to that of nitrate in order to measure the concurrent uptakes of both compounds. Nitrate concentration at the start of the incubation stage of each experiment is specified in Table 1. We did not add urea to the subculture “only Nitrate” in order to determine the nitrate uptake in the absence of urea. We used commercially available 98% ^15^N-urea, 98% ^15^N-nitrate, 98% ^13^C-bicarbonate, and 99% ^13^C-urea (Sigma-Aldrich, St. Louis, MO, USA) for tracer additions to reach final concentration of 5–10% ^15^N-urea and ^15^N-nitrate, 99% ^13^C-urea, 1% ^13^C-bicarbonate. Final concentrations were adjusted so that we would be able to measure both bulk (not too high enrichment of biomass required) and single-cell (high enrichment of cells is advantageous) samples.

**Table 1:** **Summary of data on the concurrent uptake of nitrate and urea by ***Prorocentrum minimum*****.

**Parameter**	**Experimental replicates**					
	**A**	**B**	**C**	**D**	**E**	**F**
Nitrate, μmol L^−1^	190.7 (3.3)	210.6 (3.4)	202.1 (0.4)	136.4 (3.4)	135.9 (0.9)	135.6 (1.8)
Urea addition, μmol L^−1^	100	80	80	80	80	80
Nitrate-N/Urea-N ratio	0.95	1.31	1.26	0.85	0.85	0.85
Nitrate-N uptake, fg N cell^−1^ h^−1^	309.3 (29.6)	262.2 (31.9)	369.2 (30.9)	96.6 (9.9)	98.2 (4.7)	135.6 (2.2)
Urea-N uptake, fg N cell^−1^ h^−1^	584.5 (30.3)	425.4 (13.9)	706.4 (15.8)	278.1 (7.3)	392.0 (16.9)	554.6 (44.5)
Urea-N uptake/Nitrate-N uptake ratio	1.9	1.6	1.9	2.9	4.0	4.1
% Inhibition of nitrate-N uptake	27.4	32.1	28.6	34.5	44.3	43.8

SD for each value is given in parentheses where appropriate.

Samples for isotope analysis were extracted before the addition of tracers and after the incubation lasting for 2 h. During the incubation and directly before sampling subcultures were gently agitated to ensure homogeneous conditions and cell distribution. Sampling was conducted as follows: 30 ml of each subculture were filtered onto precombusted (450°C, 4 h) glass fiber filter (GF/C, Whatman, Maidstone, UK) and washed thoroughly with isotonic NaCl solution. Following sampling, the filters with biomass were dried at 50°C overnight. The filtrates were sterilized by extra filtration through the filters with the pore size 0.2 μm (Millipore, Billerica, MA, USA) and stored at 4°C for a subsequent precise determination of nutrient concentrations. Determination of urea concentration was conducted immediately after filtration of samples in order to minimize alterations of urea content during storage. Determination of nitrate concentration was conducted within one week following the sampling. Samples from each experimental replicate were taken in duplicates. In total, six independent experimental replicates (A, B, C, D, E, and F) were performed on different days.

In order to estimate possible role of bacteria in the measured urea uptakes, before and after incubation 100–200 ml of the experimental culture were sequentially filtered using 2.7 μm pore size filters (GF/D, Whatman) to remove algal cells and precombusted filters with the pore size 0.7 μm (GF/F, Whatman) to collect bacteria. GF/F filters with bacterial biomass were processed in the same manner as described above for GF/C filters with algal biomass.

### Analytical procedures

Dissolved urea and nitrate concentrations were determined spectrophotometrically after Goeyens et al. (1998) and Doane and Horwáth (2003), respectively. For the rough and rapid determination of nitrate, we used the same protocol, but accelerated the development of color by heating the samples at 80°C for 20 min.

Cell densities were quantified using a Fuchs-Rosenthal chamber (counting at least 200 cells per sample) and a flow cytometer FACSCalibur™ (Becton, Dickinson, and Company, Franklin Lakes, NJ, USA) using a flow rate of 30 μl min^−1^, a 488 nm laser and the natural pigment fluorescence of dinoflagellates and their cell size as sorting parameters. For the cell counts, samples were fixed by a mixture of 1% formaldehyde and 1% glutaraldehyde solutions at 4°C for 30 min and then stored at −80°C until analysis.

For the determination of isotope composition of algal biomass, filters were packed into tin foil and pressed into pellets. Mass spectrometric measurements were conducted with an isotope ratio mass spectrometer Delta V (Thermo Scientific, Waltham, MA, USA) connected to an elemental analyzer via an open split interface. Reference gasses for carbon and nitrogen were ultrapure carbon dioxide and dinitrogen gas from cylinders calibrated against commercially available standards from the International Atomic Energy Agency (IAEA N1, N2, N3, C3, C6, and NBS 22). Acetanilide and Peptone (Millipore, Billerica, MA, USA) served as lab-internal elemental and isotope standards for daily calibration. The precision of the analysis was higher than 0.2o for δ^13^C and δ^15^N measurements.

### NanoSIMS analysis

Aliquots of 2–3 ml were extracted from all parallels of experimental replicates E and F and filtered onto polycarbonate filters (Millipore, Billerica, MA, USA) to yield samples with cell abundance required for NanoSIMS analysis. Filters with cells were completely dried and stored at −20°C. Before the analysis, samples were sputter coated with 30 nm gold using a 108auto Sputter Coater (Cressington, Watford, UK) to provide a conductible surface required for NanoSIMS. We randomly selected 15–25 cells from each sample and analyzed them with NanoSIMS 50L (CAMECA, Gennevilliers, France) at the Leibniz Institute for Baltic Sea Research, Warnemünde (IOW). The selected cells were inspected with the secondary electron detector of the NanoSIMS for integrity.

The NanoSIMS was tuned to detect ^12^C^−^, ^13^C^−^, ^12^C^14^N^−^, ^12^C^15^N^−^ and reach appropriate mass resolution (7485 on average, ΔM/M according to CAMECA's definition) to separate interfering masses (e.g., ^12^C^15^N^−^ from ^13^C^14^N^−^). To remove gold and reach stable ion formation conditions, an area of 50 × 50 μm was sputtered with a current of 600 pA Cs^+^ primary ions for 4 min. Analyses were performed for areas of 10 × 10 to 45 × 45 μm (depending on the number of available cells) with 1 pA Cs^+^ primary current. Sixty planes of 256 × 256 pixels were analyzed with a dwelling time of 1 ms pixel^−1^. Charge compensation was not necessary. Lateral resolution was randomly checked and better than 300 nm in all cases.

Planes were drift corrected and accumulated employing the Look at NanoSIMS software (Polerecky et al., 2012). ^13^C/^12^C or ^15^N/^14^N values and maps were obtained by taking the ratio between the ^13^C^−^ and ^12^C^−^ images or the ^12^C^15^N^−^ and ^12^C^14^N^−^ images, respectively. Isotopic ratios were calculated for regions of interest (ROIs). ROIs were drawn manually based on the cell outlines recorded by the ^12^C^14^N^−^ signal. In addition, the cell outlines were verified with the secondary electron signals detected in parallel. Random tests with prolonged cell analyses to reach deeper cell structures did not provide indication of spatial differences within cells but we admit that no analysis consumed the whole cell.

^13^C and ^15^N enrichments are expressed as a fraction of ^13^C and ^15^N, respectively, or in the delta notation (δ^13^C and δ^15^N in o) as follows:

$$\begin{array}{l} \delta^{y}X=\left(\frac{X_{mes}}{X_{nat}}-1\right)\times 1000 \end{array}$$

where *X* is the atom of interest with the nominal mass of the isotope with the lower abundance, *X*_*mes*_ is the measured isotopic ratio, *X*_*nat*_ represents the average isotopic ratio of the non-labeled control. The isotopic ratios measured for each ROI are reported in Supplementary Tables S1, S2.

### Calculations

N-specific bulk uptake rates (*V*_(**t**)_) were calculated according to the equation of Dugdale and Wilkerson (1986):

$$V_{\left(t\right)}=\;\;\frac{{}^{15}\;N_{xs}}{{(}^{15}\;N_{enr}-\langle F\rangle )\times T}$$

where ^15^*N*_*xs*_– atom% ^15^N excess in the sample, ^15^*N*_*enr*_– atom% ^15^N in the initially labeled fraction, *F* – natural abundance of ^15^N in the sample, *T* – time of incubation.

Absolute uptake rates per volume of the filtered culture (ρ_*volume*_) and per cell (ρ_*cell*_) were calculated as

$$\begin{array}{l} \rho_{volume}=\;V_{\left(t\right)}\times PN\\ \rho_{cell}=\;\rho_{volume}/d \end{array}$$

where *PN* – the particulate nitrogen on the filter, *d* – cell density in the culture.

Percent inhibition of the nitrate uptake in the presence of urea was calculated as

$$\%\;Inhibition=(1-\frac{\rho NO_{3}^{-}}{\rho NO_{3}^{-}{}_{control}})\text{*}100$$

where ρ*NO*_3_^−^ and ρ*NO*_3_${}_{control}^{-}$ the nitrate-N uptakes in the presence and absence of urea, respectively.

Coefficients of variation were calculated as

$$\begin{array}{l} CV=\frac{\sigma }{\mu } \end{array}$$

where σ − standard deviation and μ - mean value.

### Transcriptome analysis

In order to identify the putative *P. minimum* genes involved in urea and nitrate uptake, as well as the initial stages of their metabolism, we used transcriptomes and translated transcriptomes of two *P. minimum* strains (CCMP1329 and CCMP2233) available in the database of the Marine Microbial Eukaryotic Transcriptome Sequencing Project (MMETSP; http://data.imicrobe.us/project/view/104, Combined Assemblies; Keeling et al., 2014). The search of the sequences homologous to the target proteins in translated *P. minimum* transcriptomes was performed using the LocalBLASTP algorithm (BLOSUM62 matrix). The queries were obtained from the National Center for Biotechnology Information (NCBI; http://www.ncbi.nlm.nih.gov/protein) and belonged to the plant *Arabidopsis thaliana*. The *E*-value for every identified homolog was less than 10^−10^.

### Statistics

The data were analyzed with the MaxStat 3.06 software (MaxStat Software, Germany). Shapiro-Wilk test was used to test the normality of samples. Bartlett test was used to test the homogeneity of variances. Whenever possible, parametric tests (1-way ANOVA with the post-comparison Tukey test) were used. Whenever datasets did not follow the assumptions for parametric tests, non-parametric tests (Mann-Whitney test, Kruskal-Wallis test followed by the post comparison Dunn test) were used. The non-parametric Spearman rank order correlation was applied to evaluate potential relationships between variables. Differences were considered significant if *p*-values were < 0.05.

## Results

### Urea-N vs. Nitrate-N uptake and inhibition of nitrate uptake by urea at the population level

After addition of urea to the *P. minimum* culture growing on nitrate as a sole nitrogen source dinoflagellates were able to consume urea already in the first 2 h following its addition. The uptake of urea by bacteria present in the culture was less than 1% of the urea uptake by dinoflagellates and thus could be neglected.

**Figure 2:**
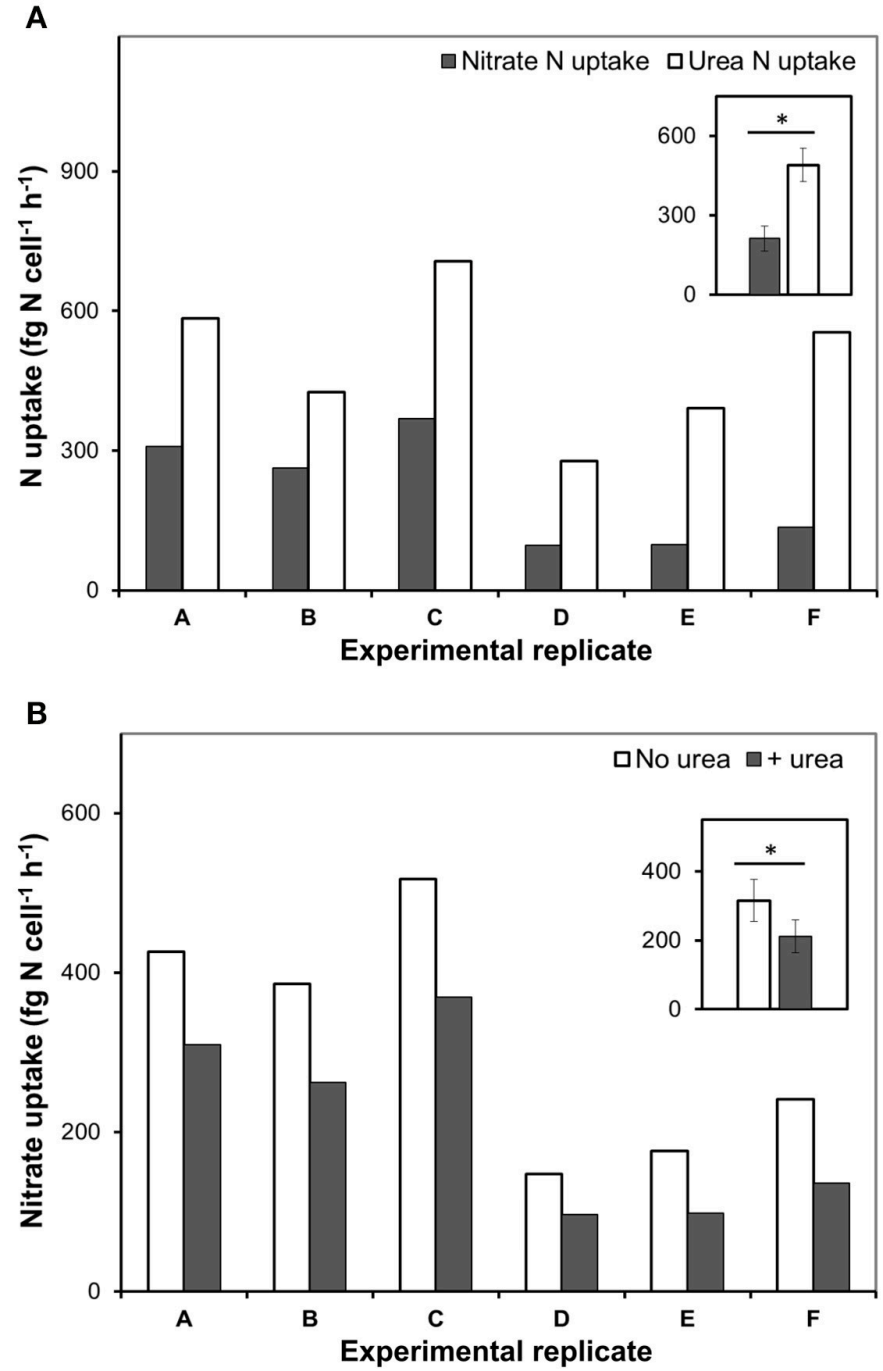
**The urea and nitrate uptake by nitrate-acclimated ***P. minimum*** following the addition of urea**. Insets show averaged data for all experimental replicates, the values are means ± 1 SE. The asterisks indicate significant differences between uptake rates. **(A)** The concurrent urea and nitrate uptake following the addition of urea. The urea-N uptake rate was significantly higher than the nitrate-N uptake rate (one-tailed Wilcoxon test, *p* < 0.05, *n* = 6). **(B)** Suppression of the nitrate uptake following the addition of urea. The nitrate-N uptake rate in the absence of urea was significantly higher than the nitrate-N uptake rate after urea addition (one-tailed Wilcoxon test, *p* < 0.05, *n* = 6).

In our experiments, rates of urea-N uptake by *P. minimum* significantly exceeded those of the nitrate-N uptake (one-tailed Wilcoxon test, *p* = 0.0156, *n* = 6) (Figure 2A, Table 1), although both nutrients were present at similar N amounts. The rates of urea-N uptake were 1.6–4.1 times higher than those of nitrate-N. Notably, the mean molar rates of nutrient uptake did not differ significantly (two-tailed Wilcoxon test, *p* = 0.4375, *n* = 6) and were 15.1 ± 3.4 fmol nitrate cell^−1^ h^−1^ and 17.5 ± 2.2 fmol urea cell^−1^ h^−1^ for nitrate and urea, respectively. The nitrate uptake rates were higher in those experiments where this N source was present at higher concentration (Experiments A, B, C, Table 1). The observed differences in the nitrate uptake rates could be attributed to different growth rates of microorganisms at the start of the incubation, since the urea and bicarbonate uptake rates also differed, even though concentrations of these nutrients were nearly the same in different experiments (Table 1).

Addition of urea to the nitrate-acclimated culture of *P. minimum* led to noticeable suppression of the nitrate-N uptake (Figure 2B, Table 1). We showed that in the presence of urea (parallels “Nitrate”) the nitrate uptake rate was significantly lower than the nitrate uptake rate in the parallels where urea was not added (parallels “only Nitrate”) (one-tailed Wilcoxon test, *p* = 0.0156, *n* = 6). We determined the urea-inhibited NO${}_{3}^{-}$ uptake as a percentage of the NO${}_{3}^{-}$ uptake in the absence of urea. Nitrate uptake was 29–44% suppressed by the presence of approximately equimolar amount of urea-N in 2 h after urea addition (nitrate-N: urea-N ≈ 1: 1). Although the ratio of nitrate-N to urea-N in the medium slightly varied from one experiment to another (Table 1), the magnitude of the observed inhibition of nitrate uptake was not related to it significantly (Spearman correlation, *r* = −0.7, *p* = 0.12, *n* = 6).

**Figure 3:**
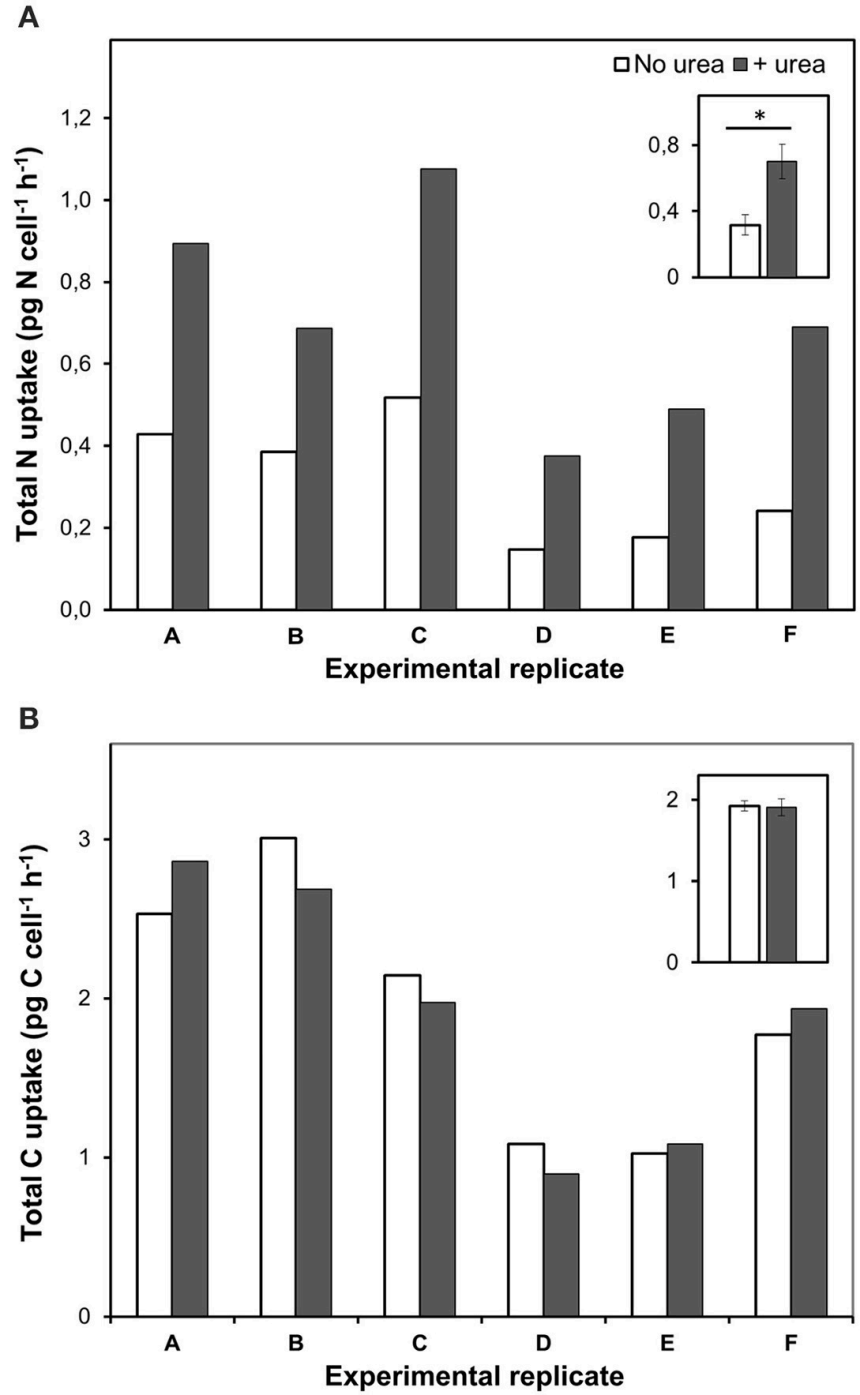
**Total nitrogen and bicarbonate uptake by ***P. minimum*** in the absence of urea and following the addition of urea**. Insets show averaged data for all experimental replicates, the values are means ± 1 SE. The asterisk indicates a significant difference between uptake rates. **(A)** Total nitrogen uptake. Total nitrogen uptake was significantly higher after urea addition (one-tailed Wilcoxon test, *p* < 0.05, *n* = 6). **(B)** Total carbon uptake. Total carbon uptake did not differ significantly in the absence and presence of urea (two-tailed Wilcoxon test, *p* = 1, *n* = 6).

Remarkably, urea addition caused a 2.4 ± 0.4-folds increase in the total-N uptake by *P. minimum* as compared to the experiments where urea was not added (Figure 3A); however, the total-C uptake (bicarbonate-C uptake plus urea-C uptake) stayed at the same level (two-tailed Wilcoxon test: *p* = 1, *n* = 6) (Figure 3B). It must be noted, that the fraction of urea-C uptake represented about 1% of the total-C uptake and was in the range of the standard deviation values for the bicarbonate uptake, which conforms to the previous data on the carbon uptake during a natural *P. minimum* bloom (Fan and Glibert, 2005).

### Single-cell uptake of urea, nitrate, and bicarbonate

**Figure 4:**
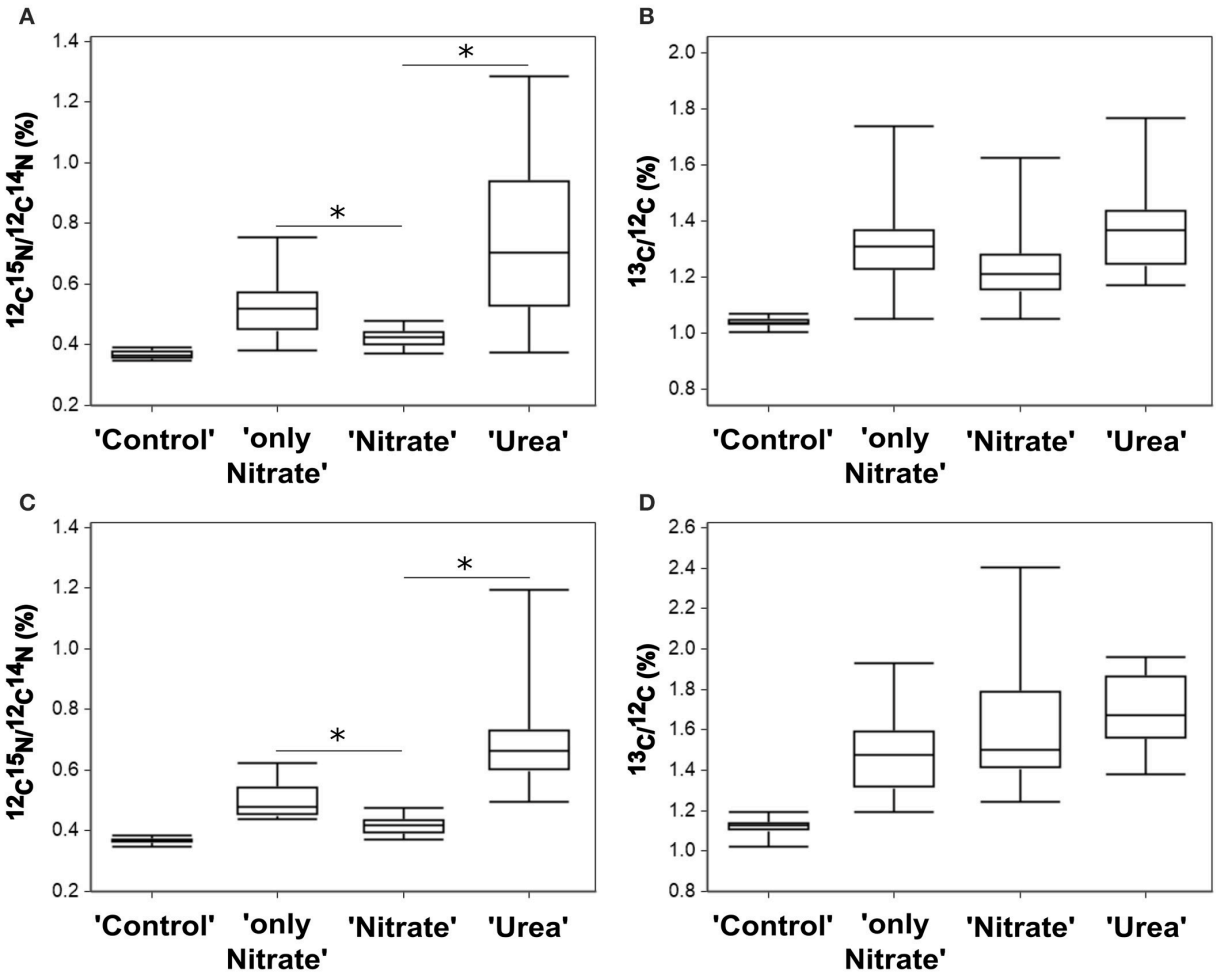
**Nutrient uptake by ***P. minimum*** measured at the single-cell level by NanoSIMS. (A)** ^15^N-enrichment in the experiment E. **(B)** ^13^C-enrichment in the experiment E. **(C)** ^15^N-enrichment in the experiment F. **(D)** ^13^C-enrichment in the experiment F. Enrichments in ^15^N and ^13^C are expressed as ^12^C^15^N/^12^C^14^N and ^13^C/^12^C, respectively. “Control”—unlabeled cells; “only Nitrate”—^15^N-nitrate and ^13^C-bicarbonate uptake in the absence of urea; “Urea”—^15^N-urea and ^13^C-bicarbonate uptake in the presence of nitrate; “Nitrate”—^15^N-nitrate and ^13^C-bicarbonate uptake in the presence of urea. In each parallel, 15–26 cells were analyzed. The horizontal lines of Box-and-Whisker plots show the median, the hinges of the box show the 25th and 75th percentiles, and the whiskers show the entire range of ^12^C^15^N/^12^C^14^N and ^13^C/^12^C ratios. Individual values are provided in Supplementary Tables S1, S2. Enrichments of all labeled parallels are significantly higher than the enrichment of “Control” cells (Kruskal-Wallis test with the post-comparison Dunn's test, *p* > 0.05). The asterisks indicate significant differences between ^15^N-enrichment of the parallels “only Nitrate” and “Nitrate” (one-tailed Mann-Whitney test, *p* < 0.001 for both experiments) and the parallels “Nitrate” and “Urea” (Kruskal-Wallis test with the post-comparison Dunn's test, *p* > 0.05; one-tailed Mann-Whitney test, *p* < 0.001 for both experiments).

**Figure 5:**
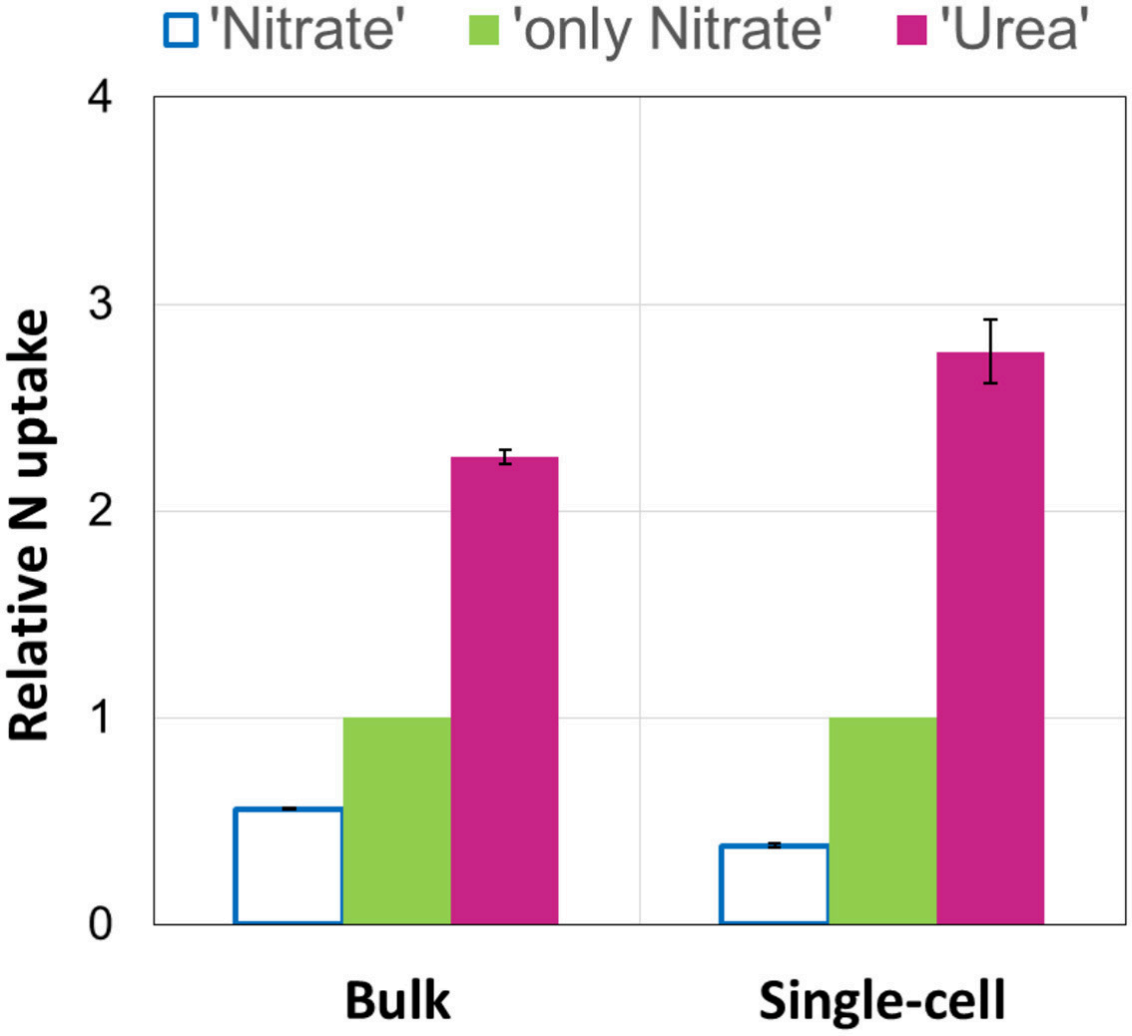
**Average relative uptake rates of urea (“Urea”) and nitrate in the presence (“Nitrate”) and absence (“only Nitrate”) of urea measured by bulk and single-cell approaches**. Nitrate uptake in the absence of urea (“only Nitrate”) was set as 1, and the concurrent uptakes of urea and nitrate (“Nitrate” and “Urea,” respectively) were expressed relative to it. The results of two experiments (E and F) are shown. The relative values are means. Bars represent the range of the individual relative values (*n* = 2). Note that the results are very similar in case of both approaches.

Using the NanoSIMS technique, we examined single-cell activities in two of the experimental replicates (experiments E and F). In total, 15–25 cells of each experimental parallel (“Urea,” “Nitrate,” “only Nitrate”), as well as non-labeled control parallel (“Control”) were analyzed. Overall, single-cell data obtained by means of NanoSIMS were in line with our bulk measurements data. In the experiments E and F, the average urea uptake rates were higher than the concurrent average uptake rates of nitrate (one-tailed Mann-Whitney test: *p* < 0.001 for both experiments); in the presence of urea the average nitrate uptake rates were lower than those in the absence of urea (one-tailed Mann-Whitney test: *p* < 0.001 for both experiments) (Figures 4A,C). Similar relationships between the relative uptake rates of nutrients determined by bulk and single-cell approaches are graphically shown in Figure 5. The average urea-N uptake rates 2.5–3 times exceeded the average concurrent nitrate-N uptake rates, and the average nitrate uptake was inhibited by ca. 40% (bulk) and 60% (single-cell) in the presence of urea.

The Kruskal-Wallis test with the Dunn post-test showed no significant difference in the average bicarbonate-C uptake rates among all labeled parallels (“Urea,” “Nitrate,” “only Nitrate”) of the experiment F and the pairs “Urea” and “Nitrate,” “Nitrate,” and “only Nitrate” of the experiment E (*p* > 0.05) (Figures 4B,D). Thus, we suggest that following the addition of urea the mean bicarbonate-C uptake rates were at the same level or very close to the initial ones, which also supports our bulk measurements.

**Figure 6:**
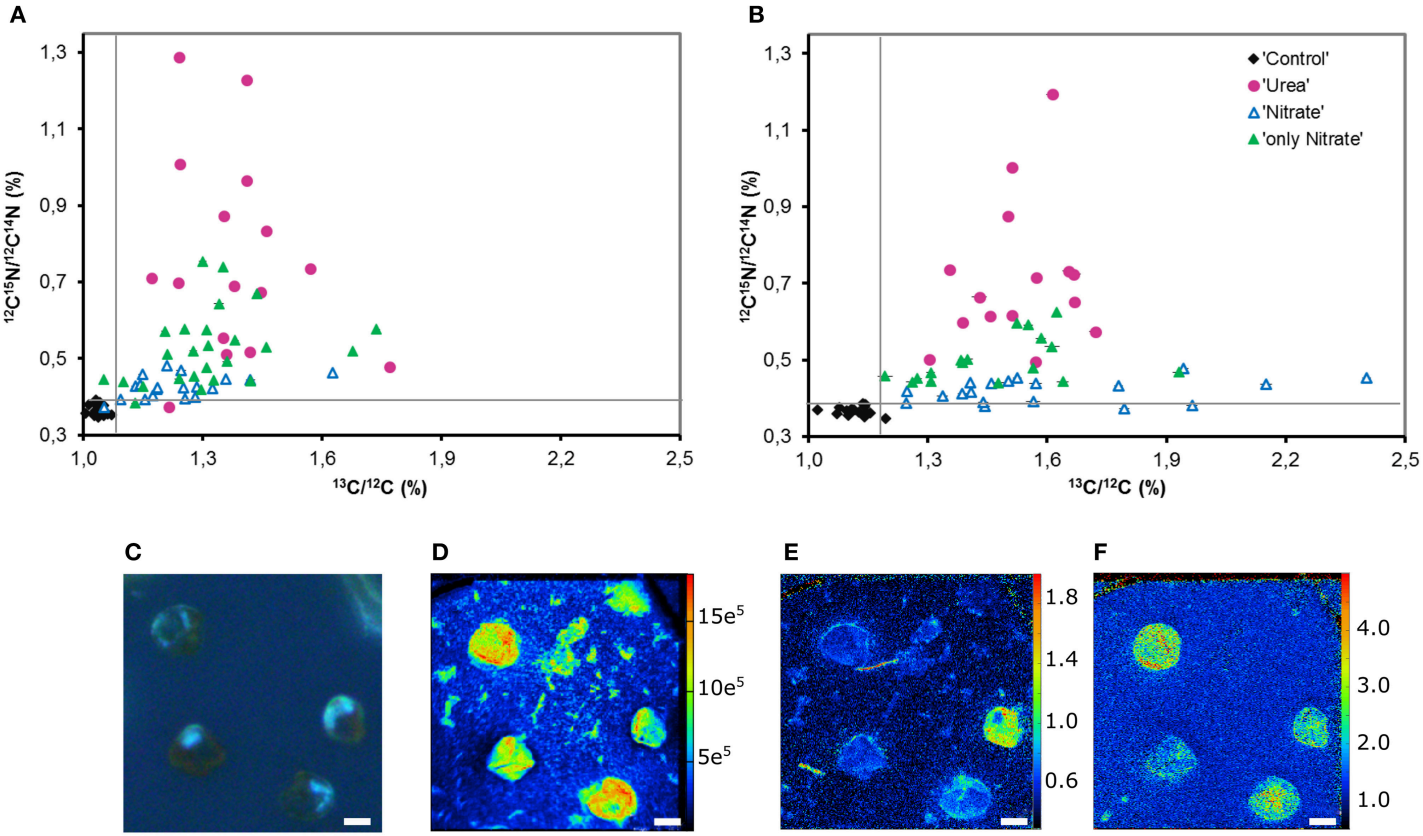
**Heterogeneity in the urea, nitrate and bicarbonate uptake by ***P. minimum*** at the single-cell level. (A)** Single-cell enrichments in ^15^N and ^13^C, expressed as ^12^C^15^N/^12^C^14^N and ^13^C/^12^C, respectively, within each parallel of the experimental replicate E. **(B)** Single-cell enrichments in ^15^N and ^13^C, expressed as ^12^C^15^N/^12^C^14^N and ^13^C/^12^C, respectively, within each parallel of the experimental replicate F (“Control”—unlabeled cells, “Urea” —^15^N-urea and ^13^C-bicarbonate uptake in the presence of nitrate, “Nitrate”—^15^N-nitrate and ^13^C-bicarbonate uptake in the presence of urea, “only Nitrate”—^15^N-nitrate and ^13^C-bicarbonate uptake in the absence of urea). **(C)** *P. minimum* cells of the parallel “Urea” in UV light. **(D)** The abundance of ^12^C^14^N^−^ in *P. minimum* cells of the parallel U depicting the cells. **(E)** ^15^N-urea uptake by *P. minimum* cells depicted as ^12^C^15^N^−^/^12^C^14^N^−^ ratio. **(F)** ^13^C-bicarbonate uptake by *P. minimum* cells depicted as ^13^C^−^/^12^C^−^ ratio. The panels **(C–F)** represent the same field of view. The abundances of masses **(C–F)** are expressed in per cents. The error bars depict Poisson error of the NanoSIMS measurements (in most cases they are so small that cannot be seen). The gray lines represent the maximal ^15^N and ^13^C atom fraction in the unlabeled parallel “Control.” Scale bar 5 μm.

Most importantly, though, NanoSIMS analysis provided the information not accessible with the use of bulk approaches. We revealed substantial variability in individual nitrogen and carbon uptake rates of distinct *P. minimum* cells within each experimental parallel (parallels “Urea,” “Nitrate,” “only Nitrate”) (Figures 4A–D, 6A–F). This variability was significant and not caused by measurement noise, which can be visually depicted by Poisson errors describing theoretical precision of measurements (Figures 6A,B). We also statistically inspected differences between cells of the least enriched parallels “Nitrate” of both experiments as recommended by Polerecky et al. (2012). We calculated the mean value and standard deviation for every cell by averaging data retrieved from all analyzed planes and then performed 1-way ANOVA with the post-comparison Tukey test in order to estimate the significance of differences between close enrichments. All calculated standard deviations were in the range of Poisson errors. The 1-way ANOVA showed that differences between individual cells of the parallels Nitrate were highly significant for ^15^N- and ^13^C-enrichments (*p* < 0.0001). The post-comparison Tukey test demonstrated that a small difference was sufficient to discriminate between two cells with a 95% confidence level: (1) a difference of 0.00635 and 0.01547% in ^15^N-enrichment; (2) a difference of 0.03607 and 0.06317% in ^13^C-encrichment for experiments E and F, respectively.

We believe that variability in single-cell enrichments disclosed by NanoSIMS was not due to the differences in size of target cells. Correlation between the size of *P. minimum* cells and their incorporation of ^15^N and ^13^C was not detected in all parallels (Spearman, −0.304 ≤ *r* ≤ 0.004, *p* > 0.05, Supporting Information Figures S1A–C), except for the parallel Nitrate where the weak negative correlation between the cell size and enrichment in ^13^C was found (Spearman, *r* = −0.383, *p* = 0.017, Supporting Information Figure S1B).

In both experiments, the absolute range of variability in stable isotope-labeled samples exceeded that of the control unlabeled samples and was approximately 9-folds in case of urea-N, almost 7-folds for nitrate and 10-folds for inorganic carbon (Figures 4A–D, 6A,B). Coefficient of variation (CV) can be used as a relative measure of variability in activity of cells. We transformed ^15^N- and ^13^C-enrichments into ^15^N- and ^13^C-uptakes by subtracting the background enrichment (mean ^15^N- and ^13^C-enrichments of the control unlabeled cells) from ^15^N- and ^13^C-enrichment values of all cells of the labeled parallels. We used obtained ^15^N- and ^13^C-uptake values to calculate CV_N_ and CV_C_, respectively, for the labeled parallels “Urea,” “Nitrate,” and “only Nitrate.” In case of ^15^N-enrichment, we determined CV_N_ of 0.61 ± 0.09 (SD, *n* = 2) for the urea uptake (parallels “Urea”), CV_N_ of 0.54 ± 0.05 (*n* = 2) for the suppressed nitrate uptake (parallels “Nitrate”) and CV_N_ of 0.53 ± 0.12 for the unsuppressed nitrate uptake (parallels “only Nitrate”). In case of ^13^C-enrichment, we calculated CV_C_ of 0.51 ± 0.17 (SD, *n* = 4) for the bicarbonate uptake in the presence of urea (parallels “Urea” and “Nitrate”) and CV_C_ of 0.54 ± 0.02 (SD, *n* = 2) for the bicarbonate uptake in the absence of urea (parallels “only Nitrate”).

We found no correlation between the rates of ^13^C- and ^15^N-enrichments of individual cells of the labeled parallels “Urea” and “Nitrate” (Spearman, *r* = −0.042, *p* = 0.824, and *r* = 0.175, *p* = 0.293, respectively; Supporting Information Figures S2A, B). Weak significant correlation between these variables was observed in the parallels “only Nitrate” of the experiment E (Spearman, *r* = 0.379, *p* = 0.013, Supporting Information Figure S2C). In other words, the cells that expressed the highest rates of N uptake often did not express the highest rates of C uptake. Remarkably, in some *P. minimum* cells spatial heterogeneity in ^15^N-enrichment was visible (Figure 6E) similar to the findings on symbiotic dinoflagellates *Symbiodinium* sp. (Kopp et al., 2013), which is an indication for N storage in cells.

Furthermore, single-cell measurements showed that inhibition of the nitrate uptake by urea was not uniform for all cells of the group “Nitrate.” ^15^N atom fraction of ca. 30% of the analyzed cells was indistinguishable from the ^15^N atom fraction of control unlabeled samples in both experiments (Figures 6A,B), which indicates complete inhibition of the nitrate uptake in these cells. It must be highlighted, that overwhelming majority of these cells remained active in terms of bicarbonate consumption. Moreover, in the presence of urea (parallel “Nitrate”) not less than 50% of cells took up nitrate at the rates comparable to the lowest rates of the nitrate uptake in the absence of urea (parallel “only Nitrate”; also ca. 50% of cells) leaving open the possibility that in some cells the nitrate uptake might be not inhibited at all. In addition, it should be taken into account that the urea-N uptake rates were not always higher than the nitrate uptake rates at the single-cell level (Figures 6A,B).

### Nitrogen and carbon uptake/metabolism genes revealed by transcriptome analysis

Scarcity of information concerning molecular basics of nutrient consumption in dinoflagellates hampers the advances in research on nutritional physiology of these organisms. In order to get some cues to interpret our data we undertook the analysis of two translated *P. minimum* transcriptomes that are publicly available (MMETSP database). It revealed the presence of sequences encoding proteins involved in uptake and the first steps of assimilatory metabolism of urea and nitrate (Table 2). We found amino acid sequences from at least two different protein families of eukaryotic origin that can putatively mediate urea transport into a cell (aquaporins MIP, selective transporters DUR3). Furthermore, we found sequences homologous to various nitrate transporters of plants (dual-affinity transporter NRT1.1, low-affinity transporter NRT1.2, and high-affinity transporter NRT2.1). We also identified the enzymes responsible for nitrate reduction to ammonium (assimilatory nitrate reductase and nitrite reductase) and for hydrolysis of urea to ammonium and bicarbonate (urease). Remarkably, the other enzyme converting urea to ammonium that is widespread among unicellular eukaryotes—urea amidolyase (Solomon et al., 2010)—was not found. The urea/amide channels and low-affinity urea transporters of the solute carrier family 14, the proteins that are known to mediate urea transport, were not detected either. Nevertheless, the absence of certain sequences in the transcriptome of *P. minimum* does not necessarily imply that these organisms lack them in their genome.

**Table 2:** **Sequences encoding putative proteins involved in nitrate and urea transport and metabolism in the transcriptome of ***Prorocentrum minimum*****.

	**Protein**	***E*-value**	**Sequence length (AA)**	**Query length (AA)**	**Coverage (%)**	**Accession number in the transcriptome CCMP2233**
Urea transport	MIP	1e^−29^	303	294	73	47098_1
	DUR3	0	1422	704	91	255379_1
Nitrate transport	NRT1.1	8e^−18^	567	590	63	258774_1
	NRT1.2	2e^−21^	701	585	89	13648_1
	NRT2.1	1e^−50^	523	530	62	255222_1
Urea and nitrate metabolism	Urease	0	814	838	97	16863_1
	Assimilatory nitrate reductase NR	0	942	917	91	257292_1
	Nitrite reductase NIR	1e^−30^	802	586	77	241489_1

## Discussion

### Concurrent uptake of urea and nitrate by *P. minimum*

The ability of nitrate-acclimated *P. minimum* to consume urea shortly after its input to the environment, even though this nutrient is new to the cells, implies the existence of a rapid and effective mechanism to recruit urea-utilizing molecular machinery. There are indications that many dinoflagellate genes involved in the essential physiological processes are regulated at the post-transcriptional level (Morse et al., 1989; van Dolah et al., 2007; Brunelle and van Dolah, 2011; Morey et al., 2011). Post-transcriptional regulation of gene expression may shorten the time required for the cellular response to various stimuli. Moreover, it is possible that proteins needed for urea utilization are permanently expressed in *P. minimum.* This assumption is supported by the data on urease activity in this species. Many studies demonstrated that a basal level of urease activity in *P. minimum* did not differ in nitrate- and urea- grown cultures (Dyhrman and Anderson, 2003; Solomon and Glibert, 2008; Liu et al., 2015). However, for a quick response to a new nutrient, cells must also carry urea transporters in their plasma membranes or be able to deliver required transporters to the plasma membrane in response to a signal.

We showed that nitrate-acclimated dinoflagellates readily consume urea at a high rate even under N replete experimental conditions; therefore, they should be able to take it up whenever it appears in much less N saturated natural habitats or in the natural ecosystems with elevated concentrations of N compounds affected by anthropogenic eutrophication. The bulk investigation conducted on natural populations of *P. minimum* (probably mixed with populations of less represented microalgae) showed that maximal uptake rates of urea-N (up to 493 fg N cell^−1^ h^−1^) always exceeded maximal uptake rates of nitrate-N (up to 341 fg N cell^−1^ h^−1^), which, to our knowledge, was the first direct estimation of the concurrent uptake of urea and nitrate by *P. minimum* in field incubations (Fan et al., 2003b). Our laboratory experiments employing monocultures of nitrate-acclimated *P. minimum* and both bulk and single-cell measurements confirm the prevalence of urea-N uptake over the concurrent nitrate-N uptake in the medium where these nutrients are simultaneously present. At the same time, the average molar uptake rates of urea and nitrate were similar, indicating that urea uptake may be advantageous in terms of energy requirements.

Interactions between concurrent uptakes of different substrates by dinoflagellates are not completely understood yet. Ammonium is known to inhibit the nitrate uptake in *P. minimum* (Lomas and Glibert, 1999; Glibert et al., 2016) and urea uptake in the dinoflagellate *Alexandrium catenella* (Jauzein et al., 2008), but no information concerning the effects of urea on the nitrate uptake was available so far. Our results represent the first evidence for partial inhibition of the nitrate uptake by urea in the dinoflagellate *P. minimum*. Presence of urea may directly trigger the suppression of the nitrate transporters. Both urea transporters DUR3 and nitrate transporters of the family NRT2 that were identified in the transcriptome of *P. minimum* act as co-transporters with protons H^+^ and thus compete for energy conserved in the proton gradient (Liu et al., 2003; Miller et al., 2007; Wang et al., 2008). It was shown that decreases in the transmembrane potential lead to the decrease in NRT2 transporter affinity in plants (Zhou et al., 1998). Thus, activation of the urea transporters can reduce the efficiency of the nitrate transport. Furthermore, the inhibitory effect of urea may actually represent the inhibitory effect of ammonium that was observed in dinoflagellates before (see above). Once urea enters a cell, it can be hydrolyzed by the enzyme urease that we also found in the transcriptome of *P. minimum*. Hydrolysis of every urea molecule produces two ammonium ions according to the urease reaction stoichiometry (Mobley and Hausinger, 1989; Mobley et al., 1995). Consequently, intracellular concentration of ammonium may be rather high in cells growing on urea, resulting in ammonium-mediated suppression of the nitrate uptake. Our findings concerning the inhibitory effect of urea on nitrate uptake by *P. minimum* are concordant with the data of Liu et al. (2015) who showed that activity of nitrate reductase in dinoflagellates *Akashiwo sanguinea* is inhibited in the presence of urea. Thus, input of urea can suppress nitrate assimilation at different levels, such as transport and reduction.

The fact that an increase in total N uptake by *P. minimum* after addition of urea was not accompanied by a simultaneous increase in total C uptake could be an evidence of an unbalanced growth response and temporary N storage in these organisms. Moreover, it cannot be ruled out that carbon required to balance uptake of extra nitrogen originated from intracellular resources, i.e. it was redistributed among different pools of C-compounds in a cell. Nevertheless, we think that N storage represents the most probable scenario. It was shown that dinoflagellates are able to store both nitrate and urea intracellularly (Flynn, 1990; Lomas and Glibert, 2000; Solomon and Glibert, 2008; Kopp et al., 2013). Importantly, such N reserves may be advantageous in habitats with episodic N supply and support growth of organisms when nutrients in the environment are exhausted (Fujita, 1985; Smayda, 1997). It should be noted that in our experiments we used high N concentrations ensuring replete N conditions that may result in nutrient storage (Flynn, 1990; Smayda, 1997). However, in natural environments luxury N status is rarely achieved; therefore, in contrast to our experiments, in natural ecosystems urea inputs are likely to enhance the uptake of inorganic carbon.

### Variability in nitrate, urea, and bicarbonate uptake of distinct *P. minimum* cells

NanoSIMS has been successfully applied to identify the functions of uncultivated microorganisms and members of mixed natural communities (Kuypers and Jørgensen, 2007; Foster et al., 2011; Krupke et al., 2013; Gao et al., 2015). Less attention has been paid to elucidating the heterogeneity within populations of the same ecologically relevant species, albeit cell-to-cell variability seems to be a universal trait of any cell population and has multiple biological consequences (Junker and van Oudenaarden, 2014).

Our single-cell enrichment data revealed different levels of nutritional activity for individual cells of *P. minimum* with similar coefficients of variation exceeding 50% in case of urea, nitrate, and bicarbonate uptake, which indicates heterogeneity of population in this respect. Previously, a large range of ammonium and inorganic carbon uptake rates for single cells of the bacterial species *Chlorobium clathratiforme* was registered in the environment (Musat et al., 2008). In that work, the observed metabolic variability was explained either by genetic heterogeneity typical for different natural populations of the same bacterial species or by non-genetic heterogeneity associated with the different microenvironments and life histories of distinct cells. In another work, the authors found pronounced heterogeneity in metabolic traits between cells of bacteria *Chlorobium phaeobacteroides* and proposed the feasibility and importance of heterogeneity studies in environmental microbiology (Zimmermann et al., 2015). In case of microalgae, variability in the rate of lipid accumulation and chlorophyll content was demonstrated on diatoms *Cyclotella cryptica*, and the researchers concluded that bulk measurements should be interpreted with caution (Traller and Hildebrand, 2013).

Variability observed among single *P. minimum* cells may be of different origin. First, not perfectly uniform conditions within batch cultures, as well as non-homogeneous distribution of stable isotope tracers, could be a reason for it. However, we tried to avoid this by thorough mixing after tracer addition and gentle agitation of subcultures during the incubation stage. Second, this variance may occur due to stochastic events during the process of gene expression, thus representing natural population variation. Research on bacterial models demonstrated that even isogenic populations cultivated in identical environment express variance for different physiological parameters at the single-cell level (Lidstrom and Konopka, 2010). Finally, heterogeneity may represent a regulated adaptive trait affected by developmental and functional states, such as age or cell cycle stage of individual cells (Elowitz et al., 2002; Martins and Locke, 2015). In any case, the observed heterogeneity in *P. minimum* must be underlain by differences in the level of expression and activity of nutrient transporters and/or by expression of different types of transporters in distinct microorganisms. The latter suggestion is corroborated by the fact that in the transcriptome of *P. minimum* we found amino acid sequences corresponding to several groups of proteins involved in the uptake of urea and nitrate (Table 2), all characterized by different affinities to the respective nutrients in plants and green algae (Galvan and Fernández, 2001; Mérigout et al., 2008; Sun et al., 2014).

There are indications that phenotypic variation becomes crucial when populations meet environmental changes (Kussell and Leibler, 2005; Acar et al., 2008). For example, heterogeneity determines the ability of some members of bacterial populations to survive severe stress factors fatal for the majority of the population (Booth, 2002). Therefore, heterogeneity is especially relevant to stability of natural microbial populations that usually persist in environments changing unpredictably. Nutritional heterogeneity of dinoflagellate populations in general and populations of *P. minimum* in particular, may represent a significant competitive advantage in natural ecosystems. On the one hand, it may keep a population prepared for the input of new nutrient sources and secure their effective utilization. On the other hand, when several different nutrient sources are available, metabolic heterogeneity may alleviate intra-population competition for resources. In our experiments, various levels of the nitrate uptake inhibition by urea and wide range of the urea uptake rates by distinct *P. minimum* cells revealed the presence of several types of cells exploiting different nutritional strategies.

### Implications for biogeochemistry and phytoplankton ecology

Our work provides one more evidence for the importance of urea for dinoflagellate nutrition. *P. minimum* populations are not only capable of rapid urea consumption, but also can reduce the rate of the nitrate uptake in response to sudden urea inputs. Moreover, under luxury N conditions they are able to store urea in internal pools (Lomas and Glibert, 2000; Solomon and Glibert, 2008; Kopp et al., 2013) that may support their growth even if ambient nutrients are exhausted (Smayda, 1997). Based on our data and findings of other researchers, we suggest that sporadic inputs of urea typical for many coastal zones are likely to affect the local N cycling and, indeed, may support the development and persistence of HABs. Rapid and effective uptake and storage/assimilation of urea by dinoflagellates may explain the development of HABs following overlooked transient urea pulses, even if measured concentration of urea in water is low during a blooming event.

**Figure 7:**
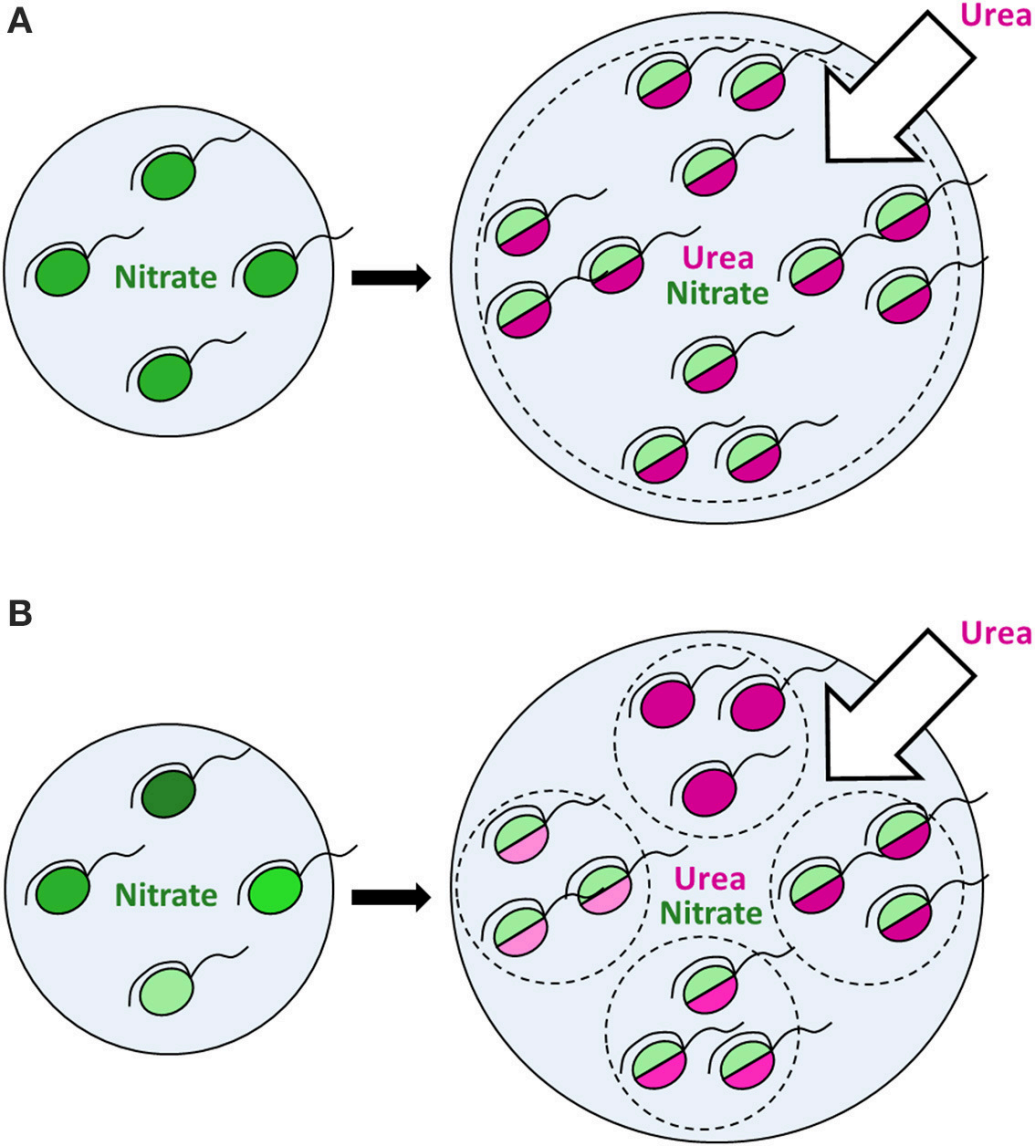
**The effect of urea input on dinoflagellate populations growing on nitrate**. Nitrogen-limited dinoflagellate populations growing on nitrate under steady-state conditions (left panels) start to consume urea and proliferate following the input of extra nitrogen in the form of urea (right panels). Green color shows the nitrate uptake, magenta—the urea uptake. The intensity of a shade reflects the uptake rate (light shades—lower uptake rates, dark shades—higher uptake rates). In a putative homogeneous population **(A)** all cells express a similar mode of nutrition with the urea uptake prevailing over the nitrate uptake and follow the same dynamics. In a heterogeneous population **(B)**, different cells express different modes of nutrition, thus forming several functional groups (sub-populations), each characterized by its own dynamics. Bulk uptake rates of nutrients might be the same in case of both homo- and heterogeneous populations at a given time, but further population dynamics can differ due to specific dynamics of each functional group within a heterogeneous population, which leads to various environmental impacts. In an utmost case, the dynamics of a heterogeneous population is defined by growth and proliferation of every individual cell, which can be described by individual-based modeling.

More intriguing, however, is that a population response does not represent just a mere sum of identical cellular responses. In contrast, it represents a superposition of various reactions of distinct cells, which should not be ignored. Since different cells express different modes of nutrition, a population comprises several groups (sub-populations), each characterized by its own fitness and growth dynamics under given conditions. This may have a large impact on dynamics of natural microbial populations, conventionally considered as homogeneous in modeling. Dynamics of a homogeneous population may significantly differ from that of a heterogeneous population due to specific dynamics of each functional group within the latter (Figures 7A,B). In an utmost case, dynamics of a heterogeneous population is defined by growth and proliferation of every individual cell. It was highlighted recently that data on heterogeneity within microbial populations should be accumulated in order to employ them in individual-based modeling of ecological processes (Kreft et al., 2013), for instance, in modeling of HABs. There are multiple studies on bacterial and mammalian cell models indicating that tiny subpopulations within seemingly homogeneous populations of cells can be of dramatic relevance under certain conditions (Altschuler and Wu, 2010). However, whether this is true or not in the case of protists of high ecological importance, i.e. phytoplankton, can only be revealed by intensive single-cell studies in this field (Matantseva and Skarlato, 2015) and application of new modeling approaches. We believe that in combination with the bulk and molecular-based techniques the single-cell research will take phytoplankton ecology to the next level.

## Author contributions

OM, SS, HS, MV designed the research; OM, IP, AV, IL performed the research; OM, AV, IP, MV analyzed and interpreted the data; OM, SS, AV, IP, IL, HS, MV, wrote the manuscript.

### Conflict of interest statement

The authors declare that the research was conducted in the absence of any commercial or financial relationships that could be construed as a potential conflict of interest.
