# Supplementary material for: Superposition of Individual Activities: Urea-Mediated Suppression of Nitrate Uptake in the Dinoflagellate Prorocentrum minimum Revealed at the Population and Single-Cell Levels
Source: Front Microbiol. 2016 Aug 25;7:1310. doi: 10.3389/fmicb.2016.01310 (PMC4996987; doi:10.3389/fmicb.2016.01310)
Supplement: Supplementary file 1 [file DataSheet1.docx]

Supplementary Material

**Superposition of Individual Activities: Urea-mediated Suppression of Nitrate Uptake by Dinoflagellates Revealed at the Population and Single-cell Levels**

**Olga Matantseva^*^, Sergei Skarlato, Angela Vogts, Ilya Pozdnyakov, Iris Liskow, Hendrik Schubert and Maren Voss**

*** Correspondence:** Olga Matantseva: matatseva@cytspb.rssi.ru

**1. Supplementary Figures and Tables**

**1.1 Supplementary Figures**


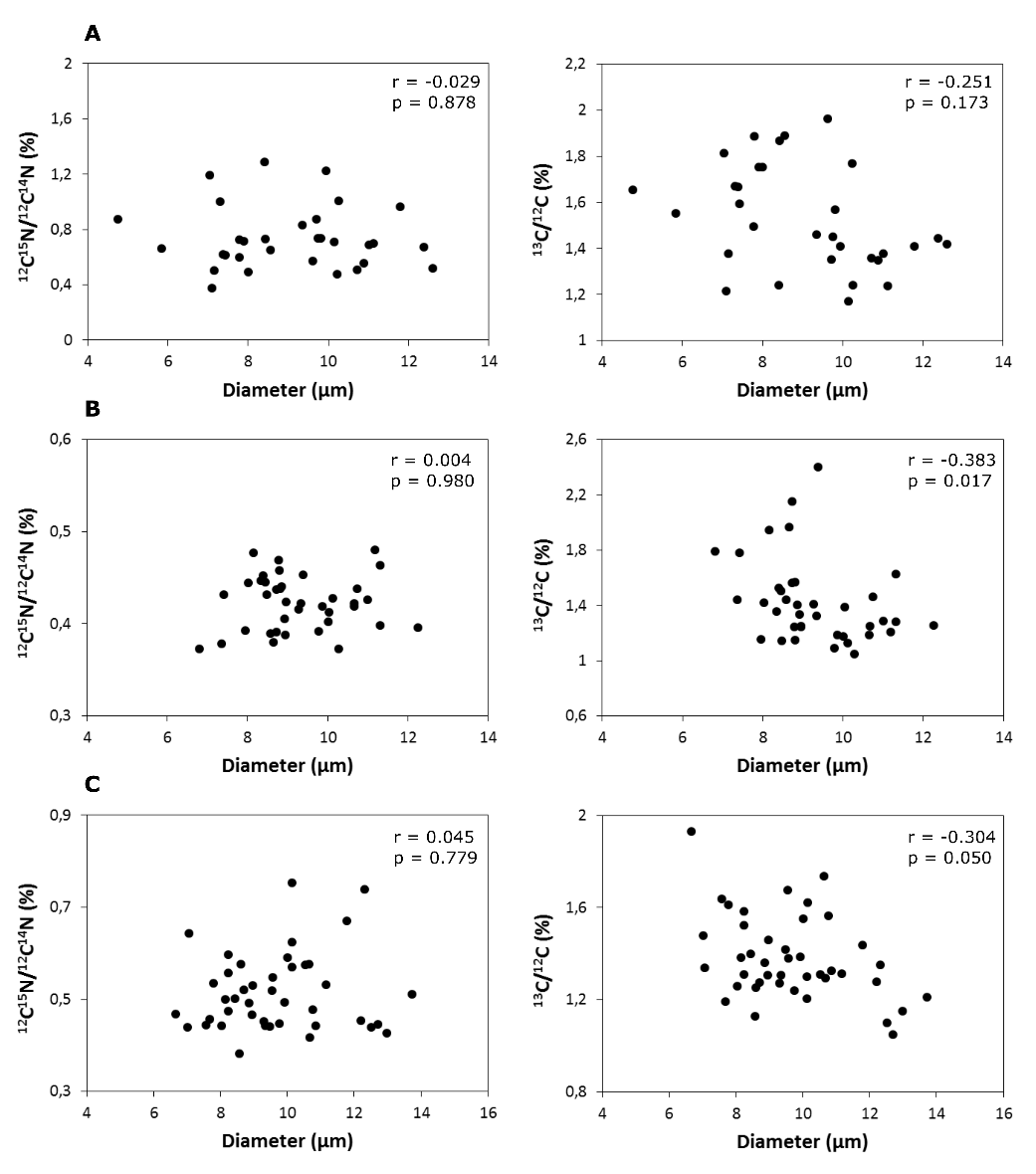


**Supplementary Figure 1.** Correlation between the cell size and ^15^N- (left panels) and ^13^C-enrichment (right panels) of *Prorocentrum minimum* cells*.* The same parallels of the experimental replicates E and F were joined. **(A)** Parallels “Urea”, **(B)** Parallels “Nitrate”, **(C)** Parallels “only Nitrate”. The size is given as a diameter of a circle with the equivalent amount of pixels as the region of interest (ROI) drawn during the processing of NanoSIMS data. Statistics on the upper right of each panel is according to the Spearman correlation test.


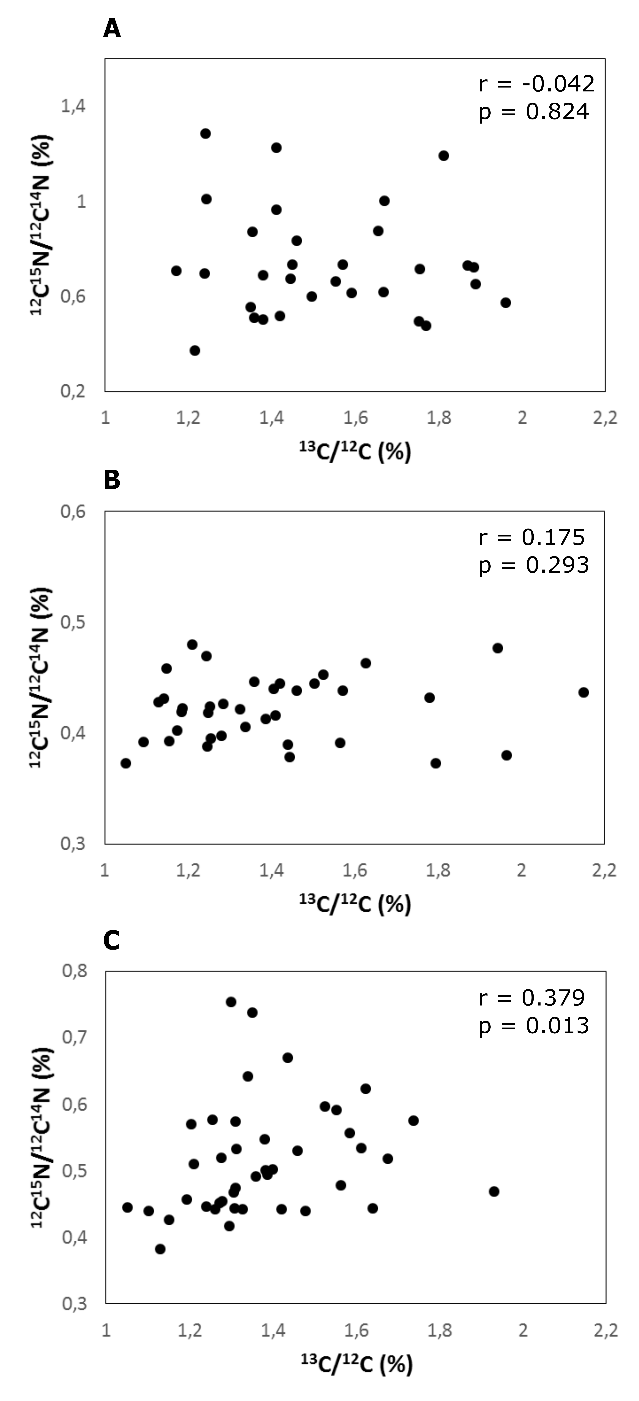


**Supplementary Figure 2.** Correlation between ^13^C- and ^15^N-enrichment of *Prorocentrum minimum* cells. The same parallels of the experimental replicates E and F were joined. **(A)** Parallels “Urea”, **(B)** Parallels “Nitrate”, **(C)** Parallels “only Nitrate”. Statistics on the upper right of each panel is according to the Spearman correlation test.

**1.2 Supplementary Tables**

**Supplementary Table 1.** The summary of raw NanoSIMS data for the experimental replicate E. Regions of interest (ROIs) refer to the analyzed cells. Additional ROIs for the filter surface were drawn for quality control of the measurements. Poisson errors are provided as given by the Look@NanoSIMS software. The size is given as a diameter of a circle with the equivalent amount of pixels as the region of interest drawn during the processing of data.

| **Sample** | **Treatment** | | **Measurement** | **ROI #** | **^13^C/^12^C value** | **Poisson error** | **δ^13^C** | **^12^C^15^N/^12^C^14^N value** | **Poisson error** | **δ^15^N** | **Size (µm)** |
| --- | --- | --- | --- | --- | --- | --- | --- | --- | --- | --- | --- |
| Exp. E  Parallel “Control” | | Non-labeled control | 160106b | 2 | 1,03E-02 | 4,18E-05 | -6 | 3,91E-03 | 7,90E-06 | 64 | 10,3 |
|  |  |  | 160106b | 3 | 1,01E-02 | 6,89E-05 | -25 | 3,79E-03 | 7,24E-06 | 31 | 11,1 |
|  |  |  | 160106c | 1 | 1,03E-02 | 4,51E-05 | -6 | 3,63E-03 | 6,96E-06 | -12 | 11,2 |
|  | |  | 160106c | 3 | 1,03E-02 | 4,66E-05 | -6 | 3,51E-03 | 6,80E-06 | -45 | 11,2 |
|  |  |  | 160106d | 3 | 1,03E-02 | 3,81E-05 | -6 | 3,59E-03 | 7,55E-06 | -23 | 10,6 |
|  |  |  | 160106e | 2 | 1,04E-02 | 6,72E-05 | 4 | 3,47E-03 | 9,24E-06 | -57 | 10,2 |
|  | |  | 160106e | 3 | 1,07E-02 | 7,33E-05 | 32 | 3,53E-03 | 6,69E-06 | -38 | 11,9 |
|  |  |  | 160107a | 2 | 1,06E-02 | 3,93E-05 | 23 | 3,54E-03 | 1,09E-05 | -37 | 10,6 |
|  |  |  | 160107a | 3 | 1,05E-02 | 5,32E-05 | 13 | 3,51E-03 | 9,69E-06 | -45 | 11,2 |
|  | |  | 160112c | 1 | 1,04E-02 | 3,47E-05 | 4 | 3,85E-03 | 8,16E-06 | 48 | 10,0 |
|  |  |  | 160112c | 3 | 1,05E-02 | 4,15E-05 | 13 | 3,77E-03 | 8,42E-06 | 26 | 11,5 |
|  |  |  | 160112d | 1 | 1,03E-02 | 3,26E-05 | -7 | 3,82E-03 | 8,41E-06 | 39 | 11,6 |
|  | |  | 160112d | 3 | 1,04E-02 | 2,64E-05 | -1 | 3,84E-03 | 7,33E-06 | 45 | 12,1 |
|  |  |  | 160112e | 1 | 1,01E-02 | 4,79E-05 | -30 | 3,57E-03 | 8,91E-06 | -29 | 9,5 |
|  |  |  | 160112e | 1 | 1,04E-02 | 4,90E-05 | 0 | 3,80E-03 | 7,77E-06 | 34 | 9,6 |
| Exp. E  Parallel “Urea” | + Urea  ^15^N-urea | | 160105a | 1 | 1,77E-02 | 5,82E-05 | 707 | 4,79E-03 | 7,19E-06 | 254 | 10,2 |
|  |  |  | 160105a | 4 | 1,17E-02 | 5,38E-05 | 131 | 7,09E-03 | 9,23E-06 | 782 | 10,1 |
|  |  |  | 160105a | 5 | 1,22E-02 | 8,50E-05 | 172 | 3,74E-03 | 1,06E-05 | 15 | 7,1 |
|  |  | | 160105b | 3 | 1,24E-02 | 4,67E-05 | 198 | 1,01E-02 | 1,13E-05 | 1466 | 10,3 |
|  |  | | 160105b | 4 | 1,45E-02 | 3,49E-05 | 394 | 6,73E-03 | 7,75E-06 | 699 | 12,4 |
|  |  | | 160105c | 2 | 1,42E-02 | 5,18E-05 | 369 | 5,17E-03 | 9,55E-06 | 341 | 12,6 |
|  |  | | 160105c | 3 | 1,38E-02 | 5,78E-05 | 331 | 6,89E-03 | 1,14E-05 | 736 | 11,0 |
|  |  | | 160105c | 4 | 1,36E-02 | 4,01E-05 | 310 | 5,11E-03 | 1,00E-05 | 328 | 10,7 |
|  |  | | 160106a | 1 | 1,46E-02 | 5,84E-05 | 409 | 8,33E-03 | 1,11E-05 | 1065 | 9,4 |
|  |  | | 160106a | 2 | 1,24E-02 | 7,34E-05 | 197 | 1,29E-02 | 1,78E-05 | 2102 | 8,4 |
|  |  | | 160106a | 4 | 1,41E-02 | 4,56E-05 | 361 | 9,65E-03 | 1,04E-05 | 1367 | 11,8 |
|  |  | | 160112a | 1 | 1,57E-02 | 7,87E-05 | 515 | 7,36E-03 | 1,38E-05 | 842 | 9,8 |
|  |  | | 160112a | 2 | 1,35E-02 | 6,41E-05 | 303 | 5,55E-03 | 1,12E-05 | 428 | 10,9 |
|  |  | | 160112a | 4 | 1,41E-02 | 5,30E-05 | 361 | 1,23E-02 | 2,01E-05 | 1966 | 9,9 |
|  |  | | 160112b | 1 | 1,35E-02 | 5,86E-05 | 306 | 8,73E-03 | 1,30E-05 | 1156 | 9,7 |
|  |  | | 160112b | 3 | 1,24E-02 | 4,65E-05 | 195 | 6,98E-03 | 1,05E-05 | 757 | 11,1 |
| Exp. E  Parallel “Nitrate” | + Urea  ^15^N-Nitrate | | 160114b | 1 | 1,16E-02 | 6,35E-05 | 119 | 3,93E-03 | 9,86E-06 | 69 | 8,0 |
|  |  |  | 160114b | 2 | 1,05E-02 | 3,96E-05 | 13 | 3,73E-03 | 1,47E-05 | 15 | 10,3 |
|  |  |  | 160114b | 4 | 1,32E-02 | 5,90E-05 | 274 | 4,22E-03 | 9,26E-06 | 148 | 9,3 |
|  |  |  | 161515a | 1 | 1,13E-02 | 8,55E-05 | 90 | 4,28E-03 | 1,31E-05 | 164 | 10,1 |
|  |  |  | 161515a | 3 | 1,21E-02 | 5,71E-05 | 168 | 4,80E-03 | 8,66E-06 | 306 | 11,2 |
|  |  |  | 161515a | 5 | 1,28E-02 | 7,31E-05 | 235 | 3,98E-03 | 9,91E-06 | 83 | 11,3 |
|  |  |  | 160115b | 1 | 1,18E-02 | 5,29E-05 | 139 | 4,02E-03 | 1,03E-05 | 94 | 10,0 |
|  |  |  | 160115b | 2 | 1,18E-02 | 7,35E-05 | 139 | 4,19E-03 | 9,31E-06 | 140 | 9,9 |
|  |  |  | 160115b | 3 | 1,25E-02 | 6,39E-05 | 206 | 4,24E-03 | 1,27E-05 | 154 | 9,0 |
|  |  |  | 160115b | 4 | 1,25E-02 | 1,14E-04 | 206 | 4,69E-03 | 1,64E-05 | 276 | 8,8 |
|  |  |  | 160115c | 1 | 1,36E-02 | 6,79E-05 | 312 | 4,47E-03 | 1,34E-05 | 216 | 8,3 |
|  |  |  | 160115c | 3 | 1,15E-02 | 9,06E-05 | 110 | 4,58E-03 | 1,15E-05 | 246 | 8,8 |
|  |  |  | 160115c | 4 | 1,14E-02 | 7,94E-05 | 100 | 4,32E-03 | 1,12E-05 | 175 | 8,5 |
|  |  |  | 160115c | 6 | 1,09E-02 | 4,66E-05 | 52 | 3,92E-03 | 1,47E-05 | 67 | 9,8 |
|  |  |  | 160115d | 1 | 1,63E-02 | 6,71E-05 | 573 | 4,63E-03 | 1,19E-05 | 260 | 11,3 |
|  |  |  | 160115d | 2 | 1,42E-02 | 1,13E-04 | 370 | 4,45E-03 | 1,42E-05 | 211 | 8,0 |
|  |  |  | 160115d | 4 | 1,29E-02 | 5,29E-05 | 245 | 4,26E-03 | 9,86E-06 | 159 | 11,0 |
|  |  |  | 160115d | 5 | 1,26E-02 | 6,97E-05 | 216 | 3,96E-03 | 9,77E-06 | 77 | 12,3 |
|  |  |  | 160115d | 6 | 1,19E-02 | 7,88E-05 | 148 | 4,23E-03 | 1,03E-05 | 151 | 10,7 |
| Exp. E  Parallel “only Nitrate” | No Urea  ^15^N-Nitrate | | 160108b | 1 | 1,21E-02 | 4,59E-05 | 168 | 5,11E-03 | 9,14E-06 | 390 | 13,7 |
|  |  |  | 160108b | 3 | 1,33E-02 | 5,80E-05 | 283 | 4,43E-03 | 8,17E-06 | 205 | 10,9 |
|  |  | | 160108b | 4 | 1,28E-02 | 5,11E-05 | 235 | 4,54E-03 | 8,45E-06 | 235 | 12,2 |
|  |  | | 160108c | 1 | 1,36E-02 | 9,55E-05 | 312 | 4,92E-03 | 1,83E-05 | 339 | 8,86 |
|  |  | | 160108c | 3 | 1,24E-02 | 9,64E-05 | 197 | 4,47E-03 | 1,80E-05 | 216 | 9,75 |
|  |  | | 160108c | 4 | 1,34E-02 | 1,13E-04 | 293 | 6,43E-03 | 1,83E-05 | 749 | 7,05 |
|  |  | | 160108c | 5 | 1,28E-02 | 9,49E-05 | 235 | 5,20E-03 | 1,29E-05 | 415 | 8,7 |
|  |  | | 160108d | 1 | 1,20E-02 | 7,73E-05 | 158 | 5,71E-03 | 9,57E-06 | 554 | 10,13 |
|  |  | | 160108d | 2 | 1,74E-02 | 7,13E-05 | 679 | 5,76E-03 | 9,77E-06 | 567 | 10,64 |
|  |  | | 160108d | 4 | 1,30E-02 | 7,67E-05 | 254 | 4,18E-03 | 8,47E-06 | 137 | 10,68 |
|  |  | | 160111a | 1 | 1,31E-02 | 6,91E-05 | 264 | 5,33E-03 | 1,27E-05 | 450 | 11,16 |
|  |  | | 160111a | 2 | 1,38E-02 | 7,94E-05 | 332 | 5,48E-03 | 1,19E-05 | 491 | 9,56 |
|  |  | | 160111a | 4 | 1,10E-02 | 6,40E-05 | 61 | 4,39E-03 | 1,30E-05 | 194 | 12,51 |
|  |  | | 160113a | 1 | 1,68E-02 | 5,80E-05 | 621 | 5,19E-03 | 8,71E-06 | 412 | 9,55 |
|  |  | | 160113a | 2 | 1,13E-02 | 7,28E-05 | 90 | 3,83E-03 | 1,56E-05 | 42 | 8,57 |
|  |  | | 160113b | 1 | 1,15E-02 | 5,31E-05 | 110 | 4,27E-03 | 9,69E-06 | 162 | 12,97 |
|  |  | | 160113b | 2 | 1,35E-02 | 6,57E-05 | 303 | 7,39E-03 | 1,36E-05 | 1011 | 12,32 |
|  |  | | 160113b | 4 | 1,30E-02 | 9,20E-05 | 254 | 7,54E-03 | 2,13E-05 | 1051 | 10,13 |
|  |  | | 160113c | 2 | 1,31E-02 | 5,23E-05 | 264 | 5,75E-03 | 1,04E-05 | 564 | 10,53 |
|  |  | | 160113c | 3 | 1,25E-02 | 6,12E-05 | 206 | 5,77E-03 | 1,40E-05 | 570 | 8,6 |
|  |  | | 160113c | 4 | 1,44E-02 | 6,31E-05 | 390 | 6,70E-03 | 1,07E-05 | 823 | 11,78 |
|  |  | | 160113d | 1 | 1,42E-02 | 6,18E-05 | 370 | 4,42E-03 | 7,51E-06 | 203 | 9,48 |
|  |  | | 160113d | 3 | 1,05E-02 | 4,56E-05 | 13 | 4,46E-03 | 6,73E-06 | 213 | 12,7 |
|  |  | | 160113c | 1 | 1,31E-02 | 4,47E-05 | 264 | 4,75E-03 | 8,74E-06 | 292 | 8,23 |
|  |  | | 160113c | 2 | 1,46E-02 | 5,47E-05 | 409 | 5,30E-03 | 8,90E-06 | 442 | 9,0 |

**Supplementary Table 2.** The summary of raw NanoSIMS data for the experimental replicate F. Regions of interest (ROIs) refer to the analyzed cells. Additional ROIs for the filter surface were drawn for quality control of the measurements. Poisson errors are provided as given by the Look@NanoSIMS software. The size is given as a diameter of a circle with the equivalent amount of pixels as the region of interest drawn during the processing of data.

| **Sample** | **Treatment** | | **Measurement** | **ROI #** | **^13^C/^12^C value** | **Poisson error** | **δ^13^C** | **^12^C^15^N/^12^C^14^N value** | **Poisson error** | **δ^15^N** | **Size (µm)** |
| --- | --- | --- | --- | --- | --- | --- | --- | --- | --- | --- | --- |
| Exp. F  Parallel “Control” | Unlabeled control | | 141119e | 1 | 1,15E-02 | 9,07E-05 | 33 | 3,94E-03 | 1,01E-05 | -14 | 10,4 |
|  |  |  | 141119e | 2 | 1,19E-02 | 9,52E-05 | 68 | 3,77E-03 | 1,19E-05 | -56 | 9,7 |
|  |  |  | 141119e | 3 | 1,14E-02 | 9,98E-05 | 24 | 3,94E-03 | 1,12E-05 | -14 | 10,0 |
|  |  | | 141120c | 2 | 1,07E-02 | 3,95E-05 | -41 | 3,91E-03 | 1,04E-05 | -21 | 11,3 |
|  |  | | 141121a | 3 | 1,10E-02 | 1,03E-04 | -9 | 4,05E-03 | 1,40E-05 | 14 | 10,8 |
|  |  | | 141121a | 4 | 1,14E-02 | 1,17E-04 | 21 | 4,13E-03 | 1,66E-05 | 32 | 8,5 |
|  |  | | 141121a | 5 | 1,10E-02 | 1,78E-04 | -17 | 3,87E-03 | 1,99E-05 | -31 | 6,8 |
|  |  | | 141121b | 2 | 1,11E-02 | 7,30E-05 | -3 | 4,02E-03 | 8,36E-06 | 7 | 9,1 |
|  |  | | 141121c | 2 | 1,12E-02 | 8,33E-05 | 8 | 3,97E-03 | 1,08E-05 | -8 | 7,3 |
|  |  | | 141211b | 2 | 1,09E-02 | 5,14E-05 | -20 | 4,00E-03 | 7,79E-06 | 1 | 8,3 |
|  |  | | 141212a | 2 | 1,07E-02 | 5,95E-05 | -37 | 4,04E-03 | 1,30E-05 | 10 | 12,3 |
|  |  | | 141212a | 3 | 1,02E-02 | 9,56E-05 | -86 | 4,19E-03 | 1,61E-05 | 48 | 9,4 |
|  |  | | 141215c | 1 | 1,14E-02 | 6,90E-05 | 22 | 3,83E-03 | 1,42E-05 | -41 | 10,0 |
|  |  | | 141215c | 3 | 1,14E-02 | 1,02E-04 | 20 | 4,20E-03 | 1,39E-05 | 50 | 7,4 |
|  |  | | 141215c | 4 | 1,13E-02 | 1,25E-04 | 16 | 4,08E-03 | 7,71E-06 | 22 | 7,9 |
| Exp. F  Parallel “Urea” | | + Urea  ^15^N-Urea | 141118a | 1 | 1,55E-02 | 1,79E-04 | 557 | 6,64E-03 | 2,22E-05 | 679 | 5,8 |
|  |  |  | 141118a | 2 | 1,59E-02 | 1,09E-04 | 606 | 6,15E-03 | 1,75E-05 | 567 | 7,4 |
|  |  |  | 141118a | 3 | 1,66E-02 | 1,24E-04 | 687 | 8,76E-03 | 2,63E-05 | 1163 | 4,8 |
|  | |  | 141118a | 4 | 1,67E-02 | 9,24E-05 | 706 | 1,00E-02 | 1,99E-05 | 1453 | 7,3 |
|  |  |  | 141118a | 5 | 1,67E-02 | 8,75E-05 | 703 | 6,18E-03 | 1,60E-05 | 573 | 7,4 |
|  |  |  | 141124a | 1 | 1,38E-02 | 1,19E-04 | 332 | 5,02E-03 | 1,63E-05 | 309 | 7,2 |
|  | |  | 141124a | 2 | 1,89E-02 | 1,71E-04 | 981 | 7,24E-03 | 1,57E-05 | 817 | 7,8 |
|  |  |  | 141124a | 4 | 1,45E-02 | 9,13E-05 | 423 | 7,36E-03 | 1,64E-05 | 843 | 9,8 |
|  |  |  | 141124a | 5 | 1,75E-02 | 1,31E-04 | 812 | 4,95E-03 | 1,56E-05 | 291 | 8,0 |
|  | |  | 141124b | 1 | 1,75E-02 | 6,99E-05 | 813 | 7,15E-03 | 1,56E-05 | 795 | 7,9 |
|  |  |  | 141124c | 1 | 1,96E-02 | 1,44E-04 | 1079 | 5,75E-03 | 1,64E-05 | 474 | 9,6 |
|  |  |  | 141124c | 2 | 1,50E-02 | 1,20E-04 | 483 | 5,98E-03 | 2,18E-05 | 528 | 7,8 |
|  | |  | 141124c | 4 | 1,87E-02 | 1,46E-04 | 960 | 7,32E-03 | 2,00E-05 | 835 | 8,4 |
|  |  |  | 141124c | 5 | 1,81E-02 | 1,56E-04 | 887 | 1,19E-02 | 3,78E-05 | 1890 | 7,0 |
|  |  |  | 141124d | 1 | 1,89E-02 | 4,41E-05 | 985 | 6,52E-03 | 8,01E-06 | 652 | 8,6 |
| Exp. F  Parallel “Nitrate” | + Urea  ^15^N-Nitrate | | 141125a | 1 | 1,24E-02 | 9,99E-05 | 114 | 4,22E-03 | 1,13E-05 | 56 | 9,0 |
|  |  |  | 141125a | 3 | 1,57E-02 | 1,50E-04 | 405 | 4,77E-03 | 1,42E-05 | 193 | 8,8 |
|  |  |  | 141125a | 4 | 1,96E-02 | 1,39E-04 | 757 | 4,14E-03 | 1,20E-05 | 36 | 8,7 |
|  |  |  | 141125b | 1 | 1,52E-02 | 1,08E-04 | 364 | 4,92E-03 | 1,31E-05 | 232 | 8,4 |
|  |  | | 141125b | 2 | 1,38E-02 | 1,07E-04 | 241 | 4,49E-03 | 1,45E-05 | 124 | 10,0 |
|  |  | | 141125b | 3 | 1,44E-02 | 1,13E-04 | 291 | 4,12E-03 | 1,26E-05 | 31 | 7,4 |
|  |  | | 141125b | 5 | 1,79E-02 | 1,15E-04 | 604 | 4,06E-03 | 1,52E-05 | 15 | 6,8 |
|  |  | | 141125c | 1 | 1,46E-02 | 8,99E-05 | 306 | 4,77E-03 | 2,03E-05 | 192 | 10,7 |
|  |  | | 141125c | 2 | 1,94E-02 | 1,22E-04 | 738 | 5,19E-03 | 1,44E-05 | 299 | 8,2 |
|  |  | | 141125c | 4 | 1,56E-02 | 1,72E-04 | 400 | 4,26E-03 | 1,46E-05 | 66 | 8,7 |
|  |  | | 141125d | 1 | 1,24E-02 | 7,64E-05 | 117 | 4,56E-03 | 1,46E-05 | 140 | 10,7 |
|  |  | | 141125d | 2 | 1,33E-02 | 1,36E-04 | 196 | 4,41E-03 | 2,38E-05 | 105 | 8,9 |
|  |  | | 141125d | 4 | 2,14E-02 | 1,47E-04 | 923 | 4,75E-03 | 2,19E-05 | 190 | 8,7 |
|  |  | | 141212b | 2 | 1,40E-02 | 8,27E-05 | 261 | 4,52E-03 | 1,86E-05 | 131 | 9,3 |
|  |  | | 141212c | 1 | 1,43E-02 | 1,15E-04 | 288 | 4,24E-03 | 1,46E-05 | 62 | 8,6 |
|  |  | | 141212c | 2 | 1,77E-02 | 9,35E-05 | 592 | 4,70E-03 | 2,22E-05 | 176 | 7,4 |
|  |  | | 141212c | 4 | 1,50E-02 | 8,66E-05 | 344 | 4,84E-03 | 2,18E-05 | 212 | 8,5 |
|  |  | | 141212c | 5 | 2,39E-02 | 1,07E-04 | 1149 | 4,93E-03 | 1,41E-05 | 234 | 9,4 |
|  |  | | 141215a | 1 | 1,40E-02 | 3,30E-05 | 258 | 4,79E-03 | 6,62E-06 | 198 | 8,9 |
| Exp. F  Parallel “only Nitrate” | | No Urea  ^15^N-Nitrate | 141126a | 1 | 1,30E-02 | 9,36E-05 | 170 | 4,82E-03 | 1,31E-05 | 207 | 9,3 |
|  | |  | 141126a | 2 | 1,58E-02 | 1,23E-04 | 418 | 6,06E-03 | 1,43E-05 | 515 | 8,2 |
|  | |  | 141126a | 3 | 1,55E-02 | 1,10E-04 | 389 | 6,43E-03 | 1,88E-05 | 609 | 10,0 |
|  | |  | 141126a | 4 | 1,40E-02 | 1,33E-04 | 252 | 5,47E-03 | 1,56E-05 | 368 | 8,4 |
|  | |  | 141126b | 1 | 1,61E-02 | 1,88E-04 | 442 | 5,82E-03 | 2,13E-05 | 456 | 7,8 |
|  | |  | 141126b | 3 | 1,63E-02 | 1,45E-04 | 466 | 4,83E-03 | 1,95E-05 | 208 | 7,6 |
|  | |  | 141126b | 4 | 1,30E-02 | 9,28E-05 | 169 | 5,08E-03 | 1,62E-05 | 272 | 8,9 |
|  | |  | 141126b | 5 | 1,47E-02 | 2,47E-04 | 322 | 4,79E-03 | 2,86E-05 | 199 | 7,0 |
|  | |  | 141126c | 1 | 1,19E-02 | 1,86E-04 | 67 | 4,98E-03 | 2,73E-05 | 246 | 7,7 |
|  | |  | 141126c | 3 | 1,27E-02 | 1,21E-04 | 138 | 4,91E-03 | 4,43E-05 | 229 | 9,3 |
|  | |  | 141126c | 4 | 1,92E-02 | 1,60E-04 | 727 | 5,10E-03 | 2,08E-05 | 276 | 6,7 |
|  | |  | 141204c | 1 | 1,38E-02 | 7,92E-05 | 237 | 5,45E-03 | 1,08E-05 | 363 | 8,2 |
|  | |  | 141204c | 3 | 1,56E-02 | 5,41E-05 | 399 | 5,20E-03 | 1,33E-05 | 301 | 10,8 |
|  | |  | 141210a | 1 | 1,52E-02 | 4,66E-05 | 363 | 6,49E-03 | 6,43E-06 | 624 | 8,2 |
|  | |  | 141210a | 2 | 1,62E-02 | 3,73E-05 | 452 | 6,79E-03 | 7,33E-06 | 699 | 10,1 |
|  | |  | 141211a | 1 | 1,26E-02 | 1,52E-04 | 127 | 4,81E-03 | 1,79E-05 | 204 | 8,0 |
|  | |  | 141211a | 2 | 1,38E-02 | 6,78E-05 | 241 | 5,38E-03 | 1,75E-05 | 345 | 9,9 |
